# Inorganic-Diverse Nanostructured Materials for Volatile Organic Compound Sensing

**DOI:** 10.3390/s21020633

**Published:** 2021-01-18

**Authors:** Muthaiah Shellaiah, Kien Wen Sun

**Affiliations:** Department of Applied Chemistry, National Chiao Tung University, Hsinchu 30010, Taiwan; muthaiah1981@nctu.edu.tw

**Keywords:** pollution, reliable nanosystems, VOC contamination, chemiresistor devices, device-based detection, sustainable application, nano-devices, diverse nanostructures

## Abstract

Environmental pollution related to volatile organic compounds (VOCs) has become a global issue which attracts intensive work towards their controlling and monitoring. To this direction various regulations and research towards VOCs detection have been laid down and conducted by many countries. Distinct devices are proposed to monitor the VOCs pollution. Among them, chemiresistor devices comprised of inorganic-semiconducting materials with diverse nanostructures are most attractive because they are cost-effective and eco-friendly. These diverse nanostructured materials-based devices are usually made up of nanoparticles, nanowires/rods, nanocrystals, nanotubes, nanocages, nanocubes, nanocomposites, etc. They can be employed in monitoring the VOCs present in the reliable sources. This review outlines the device-based VOC detection using diverse semiconducting-nanostructured materials and covers more than 340 references that have been published since 2016.

## 1. Introduction

Volatile organic compounds (VOCs) are the well-known cause of indoor pollution, which can harm the human health and leads to various disorders [1,2,3]. Long-time exposure to VOCs, such as phosgene, can lead to death and severe chronic diseases [4,5]. Another example is Benzene (a well-known carcinogen), which have the great potential to damage human tissues such as spleen, stomach, liver, kidneys, etc., and also affect the nervous, circulatory, immune, cardiovascular, and reproductive and respiratory systems [6,7]. Thus, to overcome such potential hazards of VOCs, numerous environmental safety agencies like Environmental Protection Agency (EPA), National Institute of Occupational Safety and Health (NIOSH), and European Agency for Safety and Health at Work (EU-OSHA), have fixated the accepted limit of certain VOCs down to sub-ppb concentrations [6]. To quantify such harmful VOCs, researchers focused to develop various sensory materials and to achieve the signals by means of fluorescent, electrochemical, or current–voltage (I-V) fluctuations, etc. [6,7,8,9,10]. Wherein, with the uniqueness and high sensitivity, chemiresistor device-based VOCs detection seems to be an interesting research topic [11,12]. In this track, nanomaterial-based semiconducting chemiresistor devices for VOCs monitoring are impressive in terms of sensitivity and selectivity [13,14].

Materials with internal or external dimensions at nanoscale (<100 nm) are defined as nanomaterials [15,16]. They can be available in one to three dimensions (1D to 3D) with diverse structures like nanoparticles, nanocrystals, nanowires, nanocubes, nanosheets, nanotubes, nanosheets, nanocages, etc. [17]. Those nanostructured semiconducting materials play a vital role in sustainable applications such as conductivity studies, transistors, solar cells, healthcare diagnostics, sensors, and so forth [18,19,20,21,22]. Among them, utilization of semiconducting nanomaterials-based sensing of VOCs/gases seems to be more attractive [23,24]. Therefore, many reviews are available on 1D to 3D inorganic-nanomaterials-based semiconducting gas/VOCs sensors [25,26,27,28,29]. Similarly, the utilization of semiconducting polymeric/hybrid/organic-based semiconducting nanomaterials were also proposed towards selective sensing of VOCs [30,31,32,33,34,35,36,37]. However, contrast to those polymeric/hybrid/organic nanostructures, inorganic semiconducting nanomaterials are highly admirable due to the low cost processing, durability, environment affordability, and high reproducibility, etc. [38,39,40]. Therefore, the effects of diverse nanostructured properties of inorganic semiconducting materials towards the determination of VOCs require more discussions. For example, Tin dioxide (SnO_2_) seems to exist in diverse nanostructured forms, like nanoparticles, nanowires, hollow micro/nano-spheres, nanofibers, and cubahedra, with specific selectivity to different VOCs, such as acetone, ethanol, butanol, and toluene [38,39,40,41,42,43]. Thus, it is essential to gather information on consumption of diverse nanostructured materials in the device based detection of VOCs.

To this path, reviews on analytes quantification by means of electrochemical devices have already been reported by many researchers [44,45]. However, the information regarding the utilization of diverse inorganic-nanostructures in chemiresistor device-based VOCs determination, which is necessary for upcoming researchers, is currently deficient. Therefore, we deliver a comprehensive review on diverse nanostructured semiconducting nanomaterials that has been employed in device-based VOCs quantifications based on recent reports (more than 340 references published since 2016).

**Figure 1:**
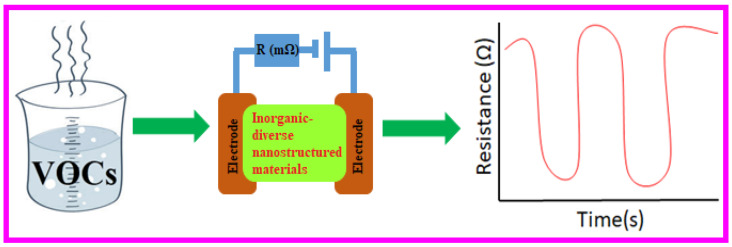
Schematic illustration of device based sensing of diverse nanostructures to volatile organic compounds (VOCs).

In this review, valuable information on diverse nanostructures employed in chemiresistor device-based VOCs detection and quantification (Figure 1) is revealed and discussed. Moreover, the mechanisms and effects of semiconducting nanostructures are clarified with charge/electron transport properties of those sensory nanomaterials. Lastly, synthesis of diverse nanostructured materials, advantages and disadvantages of those nanostructures, and their future scope towards VOCs quantification are summarized with justification.

## 2. Materials and Diverse Nanostructure Selections

In general, design and development of semiconducting nanomaterial-based gas sensors need to follow some important rules as follows.

- For device-based detection, the materials must have the unique p- or n-type semiconducting properties, which can be further boosted by combining with other materials to form a p-n or p-p or n-n heterojunction towards specific analyte quantification [46].
- The selected nanomaterials must have large surface to enhance the adsorption of gaseous VOCs, which can induce signal responses, such as I-V fluctuations, electrochemical responses, fluorescent deviations, etc. However, for device-based sensing, signals are obtained as either I-V or electrochemical responses.
- To attain the discrimination of diverse VOCs, different 1D to 3D nanostructures (nanoparticles/quantum dots, nanocrystals, nanorods/nanowires/nanoneedles, nanofibers/nanobelts, nanotubes, nanocubes, nanocages, nanowalls, nanosheets, nanoflakes/nanoplates, nanospheres, nanoflowers, porous-nanostructures, hierarchical nanostructures, and so on) can be adapted via optimizing their selectivity and sensitivity to specific analyte [47,48,49,50,51,52,53,54,55,56,57,58].
- Diverse nanostructures can be developed by chemical synthesis, hydrothermal, chemical vapor deposition (CVD), combustion synthesis, sputtering, electrospinning, impregnation, sol–gel, solid-state reaction, hybrid composite synthesis, etc., to achieve specific sensitivity to the target VOC [47,48,49,50,51,52,53,54,55,56,57,58]. However, this should be done without affecting semiconducting properties of the proposed nanostructure, otherwise the sensitivity and reproducibility may be affected significantly.
- Fabricated nanostructures on device must withstand different VOC exposures and at diverse humid/temperature conditions. Many devices may be affected by humidity of the environment and lead to malfunction, thereby special attention should be paid to the effect of humidity on the sensory devices. Similarly, certain semiconducting/hybrid materials can function at either higher or lower temperatures. Therefore, justification of operating temperature is a must for the sensory devices.

## 3. Diverse Nanostructures in Acetone Detection

Detection of acetone vapor by diverse nanostructures and materials follows the general mechanism of adsorption of volatile vapor over the surface of the substrate, resulting to the release of electrons and induce resistance changes (R_a_/R_g_; R_a_ and R_g_ are resistance without and with the target gas). During the injection of acetone vapor in the gaseous chamber consist of diverse nanostructured materials, the vapor reacts with the oxygen species that is already adsorbed, as descripted in the Equations (1) and (2), which releases electrons to be detected as sensor signal (R_a_/R_g_).

(CH_3_COCH_3_)_g_ → (CH_3_COCH_3_)_ads_ (1)

(CH_3_COCH_3_)_ads_+ 8O^−^ → 3CO_2_ + 3H_2_O + 8e^−^ (2)

The sensitivity can be varied depending on the adsorption surface area, which might be the concern with diverse nanostructures towards VOCs detection. Such quantifications can also be significantly affected by the semiconducting properties, such as p-type, n-type, p-n, p-p, and n-n heterojunctions. Thus, information on miscellaneous nanostructured materials that has been reported in acetone detection and sensitivities are outlined below.

**Figure 2:**
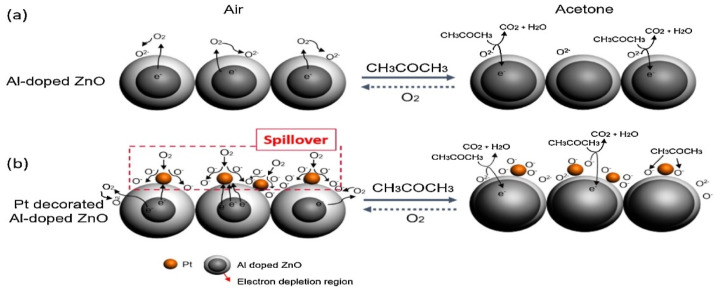
Schematic illustrating the sensing reaction mechanism of (**a**) Al-doped ZnO (AZO) and (**b**) Pt-decorated AZO (Pt-AZO) sensors in air and acetone (Reproduced with the permission from Ref. [62]).

Among various nanostructures, the semiconducting nanoparticles (NPs) are capable of providing uniform surface for acetone adsorption to deliver unique resistance changes at certain temperatures. To this path, TiO_2_ NPs, α-Fe_2_O_3_ NPs, Mn@ZnO NPs (MZO NPs; p + n interlock, field effect transistor), Pt-decorated Al-doped ZnO (Pt-AZO NPs), AZO NPs, B-TiO_2_@Ag NPs, La_1-x_Y_x_MnO_3-⸹_ NPs, and Bi_1-x_La_x_FeO_3_ NPs were consumed in selective device-based quantification of acetone with part per billion/parts per million (ppb/ppm) detection limits (LODs) [59,60,61,62,63,64,65,66]. These nanoparticles are generally synthesized via hydrothermal (TiO_2_ NPs, MZO NPs, Pt-AZO NPs, AZO NPs, and B-TiO_2_@Ag NPs), reverse micro-emulsion (α-Fe_2_O_3_ NPs), and sol–gel (La_1-x_Y_x_MnO_3-⸹_ NPs and Bi_1-x_La_x_FeO_3_ NPs) methods that operate between 250 and 500 °C (as summarized in Table 1). Among these nanoparticles, Pt-AZO NPs are quite unique with a R_a_/R_g_ response of 421, due to the doping and decoration of Al and Pt, correspondingly. Wherein, decoration of Pt enhances the sensor responses via increased O_2_ adsorption (which reacts with acetone to release more electrons) on the surface, as shown in Figure 2. However, the main drawback of this work is its operating temperature (450 °C).

**Figure 3:**
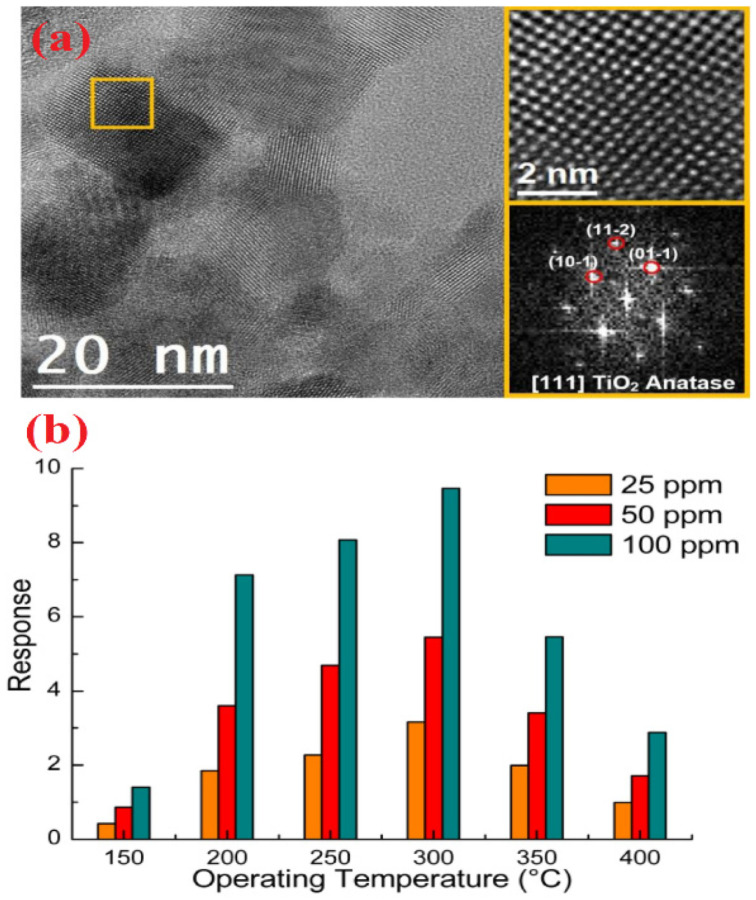
(**a**) HRTEM micrograph of the 400 °C TiO_2_-5Rh sample, detail of the orange squared region and its corresponding power spectrum; (**b**) Comparison between the TiO_2_-5Rh acetone responses at various operating temperatures. The typical volcano behavior can be observed (Reproduced with the permission from Ref. [69]).

Similar to NPs, nanocrystalline materials were also involved in volatile acetone discrimination. For example, Zhang et al. developed the Pd-doped SmFe_0.9_Mg_0.1_O_3_ nanocrystals by a sol–gel tactic and utilized in acetone detection under light illumination at 220 °C [67]. Wherein, the Pd:SmFe_0.9_Mg_0.1_O_3_ displayed an impressive response of 7.16 (for 0.5 ppm) with a LOD of 0.01 ppm. To this track, hydrothermally synthesized WO_3_ nanocrystals were also displayed a good response (R_a_/R_g_ = 3.8; 0.25 ppm) at 320 °C with a satisfactory LOD of 0.075 ppm [68]. Moreover, Rhodium (Rh) additive in the solvothermally synthesized TiO_2_ nanocrystals (TiO_2_-5Rh) slightly improved the sensor response (R_a_/R_g_ = 9.6; 50 ppm) at 300 °C, as shown in Figure 3 [69]. However, this work still needs further optimization on the operating temperature and LOD. Considering the acetone quantitation, nanowires (NWs) were fabricated to afford device-based sensors. Functionalization or attachment of certain materials on the surface of NWs may enhance the sensitivity.

**Figure 4:**
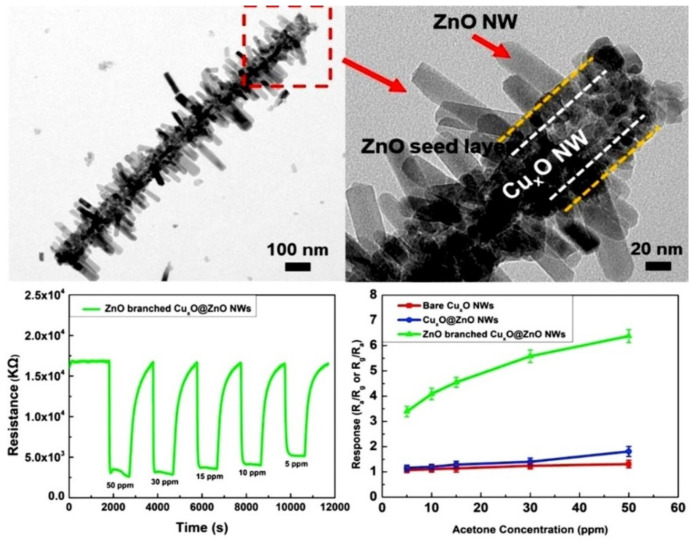
TEM image of ZnO branched p-CuxO @n-ZnO heterojunction nanowires and its sensor responses to acetone (Reproduced with the permission from Ref. [72]).

To this light, Kim et al. and Singh et al. demonstrated an exceptional acetone sensitivity (refer to Table 1) of Co_3_O_4_ NPs modified SnO_2_ NWs and self-assembled monolayer (SAM) functionalized ZnO NWs [70,71]. These sensory materials can be synthesized via vapor–liquid–solid (VLS), sol–gel, and thermal annealing processes to detect acetone with an LOD of 0.5 ppm. Additionally, p-n heterojunction NWs were proposed with ZnO branched p-CuxO@n-ZnO NWs (fabricated by hydrothermal method and atomic layer deposition) for acetone sensing [72]. Wherein, the sensor operated at 250 °C and displayed low sensor responses of 3.39–6.38 (5–50 ppm), as depicted in Figure 4. Therefore, the device requires further optimization to attain a higher response to achieve excellent performance.

Discrimination of acetone was also demonstrated by hydrothermally/chemically synthesized nanorods (NRds) and nanoneedles at certain operating temperatures with good response/recovery time, as seen in Table 1 [73,74,75,76,77]. Among these nanorods (Cr doped ZnO NRds, SnS_2_ NRds, Au/Pd-doped ZnO NRds, and α-Fe_2_O_3_/NiO NRds), the α-Fe_2_O_3_ NPs-doped NiO NRds [76] displayed a high response of 290 for 100 ppm acetone at 280 °C with a LOD of approximately 5 ppm. This might be due to its p-n heterojunction property, but the operation temperature of the sensor operation requires further reduction. Because the Ag-doped ZnO nanoneedles [77] did not display any exceptional response (R_a_/R_g_ = 30.233; for 200 ppm at 370 °C); therefore, they did not play an important role in volatile acetone detection. Devices with hydrothermally synthesized nanoarrays, such as La-doped SnO_2_, hybrid 1D/2D α-Fe_2_O_3_-SnO_2_, and ZnTiO_3_ were engaged in acetone gas estimation [78,79,80]. Wherein, in contrast to other nanoarrays, ZnTiO_3_ nanoarrays [80] showed exceptional sensor responses of 78/94 for 12.5 ppm at 45 °C/350 °C with the LODs of 0.09 and 0.01 ppm, correspondingly. This work is impressive because its operation at dark and light conditions can be completed with less than 3 min response/recovery time.

Volatile acetone quantitation was also authenticated by many semiconducting nanofibers fabricated by hydrothermal, electrospinning, and calcination tactics. Nanofibers like Ag-decorated SnO_2_, PrFeO_3_, Pt-ZnO-In_2_O_3_, Au@WO_3_-SnO_2_, In-doped ZnSnO_3_, ZnO, and Ru-doped SnO_2_ showed selective sensitivity to acetone at various operating temperatures (150–300 °C) [81,82,83,84,85,86,87]. Among them, Au@WO_3_-SnO_2_ [84] developed by Shao et al. displayed a high response of 196.1 for 10 ppm at 150 °C, with an estimated LOD of <0.5 ppm and response/recovery time of <2 min. In this track, functionalized/sensitized/assembled/decorated nanotubes (NTs) and multi-walled carbon nanotubes (MWCNTs) were reported by many research groups towards acetone sensing. Pd/Pt functionalized SnO_2_ NTs, PdO@ZnO−SnO_2_ NTs, α-Fe_2_O_3_ NRds-MWCNTs, Co_3_O_4_—MWCNTs, Pt-CuFe_2_O_4_ NTs, Pd@WO_3_–SnO_2_ NTs, and ZnO-Decorated In/Ga Oxide NTs (synthesized by hydrothermal, encapsulation, electrospinning, and soaking) were reported for exceptional acetone detection at various operating temperatures (225–400 °C) [88,89,90,91,92,93,94]. These decoration of certain catalytic substrates may enhance acetone selectivity by varying the operating temperature. For example, Pt or Pd loaded multidimensional SnO_2_ NTs [88] displayed an exceptional sensitivity to acetone (R_a_/R_g_ = 93.55 for 5 ppm at 350 °C) with a LOD of <1 ppm. Similarly, PdO−ZnO composite on hollow SnO_2_ NTs [89] showed a good sensor response to acetone even at low concentration (Rair/Rgas = 5.06 for 1 ppm at 400 °C) with a LOD of 0.01 ppm. This sensor operates under higher humidity (95% RH) than that of others, thereby is quite noticeable. The sensitivity can be enhanced by forming multiple heterojunctions and by chemical sensitization effect of nanocatalyst.

For instance, the sensitivity of Pd@WO_3_–SnO_2_ NTs [93] to acetone was enhanced by multiple heterojunctions formation (W_O3_–SnO_2_ n–n junctions, PdO–SnO_2_, and PdO–WO_3_ p-n junctions) and by the sensitization effect of Pd nanocatalyst. Doped SnO_2_ nanobelts were employed as sensor materials for the discrimination of acetone with LODs down to sub-ppm level [95,96]. Li et al., and Chen et al. reported the development of Y- or Eu-doped SnO_2_ nanobelts by thermal evaporation tactic above 1350 °C and utilized them in acetone sensing with verified enhanced performance (refer to Table 1) than those of pure SnO_2_ nanobelts. Volatile acetone gas detection has been well demonstrated by nanocubes derived from p-type Co_3_O_4_, hybrid In_2_O_3_@RGO, Ag functionalized ZnSnO_3_, ZnO−CuO p-n heterojunction, p-type NiFe_2_O_4_, NiO/ZnO composite, and MOF derived-ZnO/ZnFe_2_O_4_ [97,98,99,100,101,102,103]. Among them, ZnO−CuO p-n heterojunction displayed a good sensor response of 11 to 1 ppm acetone at 200 °C with an impressive LOD of 0.009 ppm. Moreover, the material has a stable response up to 40 days, thereby becomes a notable candidate. Similarly, hybrid In_2_O_3_@RGO nanocubes [98] showed discriminative sensing to acetone and formaldehyde at 175 °C and 225 °C, respectively, but the interference studies were still lacking in this report.

**Figure 5:**
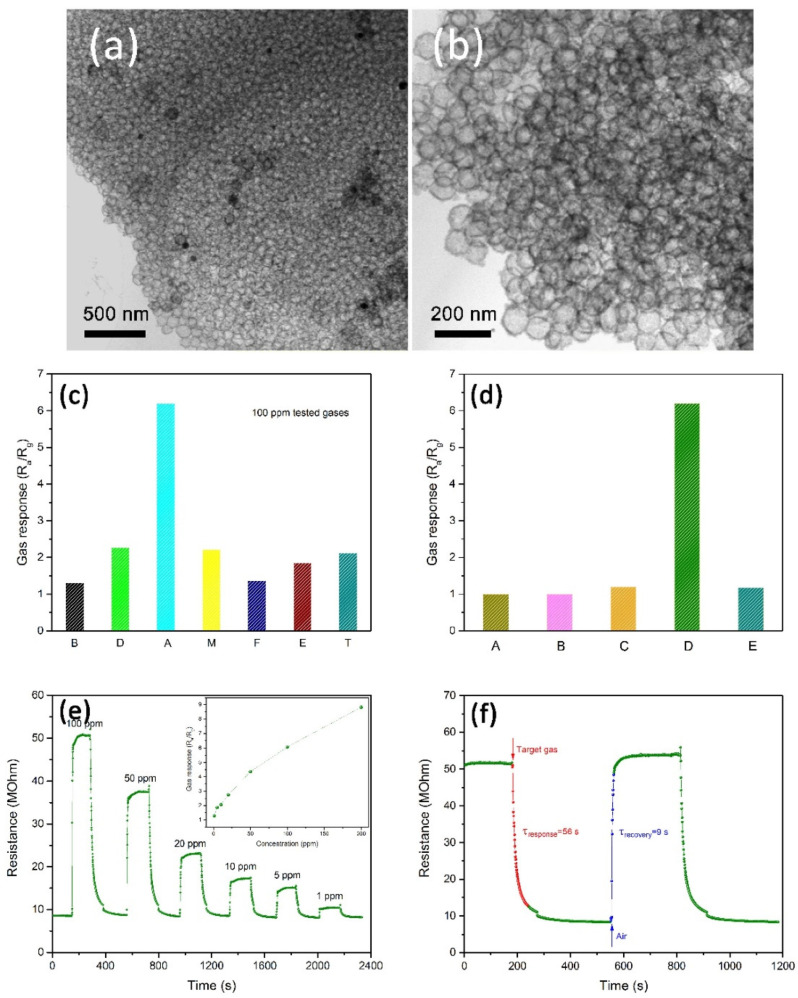
(**a**,**b**) The TEM images of Ag@CuO-TiO_2_ hollow nanocages; (**c**) Gas responses to 100 ppm various target gases at 200 °C (B, benzene; D, dimethylbenzene; A, acetone; M, methanol; F, formaldehyde; E, ethanol; T, toluene); (**d**) Gas responses of different compositions to 100 ppm acetone at 200 °C (A–D, A: Ag@CuO; B: TiO_2_; C: CuO-TiO_2_; D: Ag@CuO-TiO_2_) and 375 °C (E: TiO_2_) (**e**) Responses vs. acetone concentrations at 200 °C; (The inset figure is the linear relationship between response and concentration) (**f**) Response and recovery curves to 100 ppm acetone at 200 °C (Reproduced with the permission from Ref. [107]).

The acetone sensing capability of nanocages has been established by PdO functionalized Co_3_O_4_ hollow nanocages, ZnO/ZnFe_2_O_4_ hollow nanocages, PdO functionalized NiO/NiCo_2_O_4_ truncated nanocages, and Ag@CuO-TiO_2_ hollow nanocages [104,105,106,107]. Wherein, PdO acted as a catalyst (in PdO functionalized Co_3_O_4_ hollow nanocages and PdO functionalized NiO/NiCo_2_O_4_ truncated nanocages) to enhance the sensitivity and PdO@Co_3_O_4_ hollow nanocages [104] displayed a response of 2.51 to 5 ppm acetone at 350 °C with a LOD of 0.1 ppm. Moreover, PdO@Co_3_O_4_ hollow nanocages can be operable at 90% humid condition. Next, ZnO/ZnFe_2_O_4_ hollow nanocages [105] also detect the acetone gas to certain extend (R_a_/R_g_ = 25.8 for 100 ppm at 290 °C), but require optimization on operating temperature. On the contrary, multicomponent Ag@CuO-TiO_2_ hollow nanocages [107] enhance the acetone sensing at low operating temperature (R_a_/R_g_ = 6.2 for 100 ppm at 200 °C) with a calculated LOD of ~1 ppm, as illustrated in Figure 5. However, more focus is required to improve the response.

**Figure 6:**
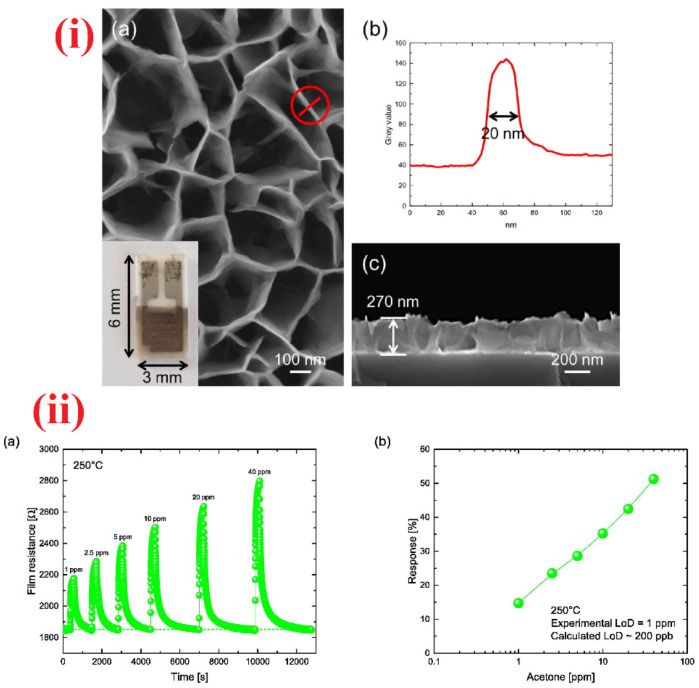
(**i**) (**a**) Plan-view SEM image of the NiO nanowalls (the inset shows the NiO-based sensor), (**b**) grey value profile along the line perpendicular to the nanosheet marked with the red circle, and (**c**) cross-view SEM image of the NiO nanowalls; (**ii**) (**a**) Dynamic responses of the NiO-based sensor to acetone pulses in the concentration range 1–40 ppm at 250 °C and (**b**) calibration curve at 250 °C (Reproduced with the permission from Ref. [115]).

Researchers synthesized various nanosheets (NShs) by means of post-thermal, hydrothermal, impregnation, liquid exfoliation, precipitation, and multistep approaches and applied them in gaseous acetone quantification. Co_3_O_4_ NShs, ZnO NShs, SnO_2_/Fe_2_O_3_ multilayer NShs, NiO NShs, and F-doped TiO_2_ NShs were engaged in acetone sensing at different operating temperatures as summarized in Table 1 [108,109,110,111,112]. In particular, F-doped TiO_2_ NShs grown on Ti foam [112] function linearly in the detection range of 25–800 ppm at 25 °C and are stable at divers humid conditions (20–90% RH), thereby become exceptional material for acetone detection. Subsequently, materials with nanowalls were utilized in acetone sensory, which showed the unique advantages of wide surface area for volatile gas adsorption. Nb-doped ZnO nanowalls (synthesized by radio-frequency (RF) magnetron sputtering), CuO nanowalls (from Oxidation of Cu foil in aqueous NH_4_OH), NiO nanowalls (from chemical bath deposition (CBD), and ZnO deposited carbon nanowalls (from microwave plasma-enhanced chemical vapor deposition (MPECVD) were reported as acetone sensors with extensive selectivity [113,114,115,116]. Herein, Nb-doped ZnO nanowalls [113] operate at 200 °C and deliver a high response of 89.13 (for 100 ppm) with good linear response between 20 and 100 ppm. Moreover, NiO nanowalls [115] exhibit exceptional response to acetone (R_a_/R_g_ ≥ 30; for 10 ppm at 250 °C) with a LOD of 0.2 ppm and exhibit the dynamic response as shown in Figure 6. Similarly, Choi et al. established wide surface area interaction of acetone to ZnO deposited carbon nanowalls [116]; therefore, development of such nanowalls-based VOCs sensors are highly anticipated.

**Figure 7:**
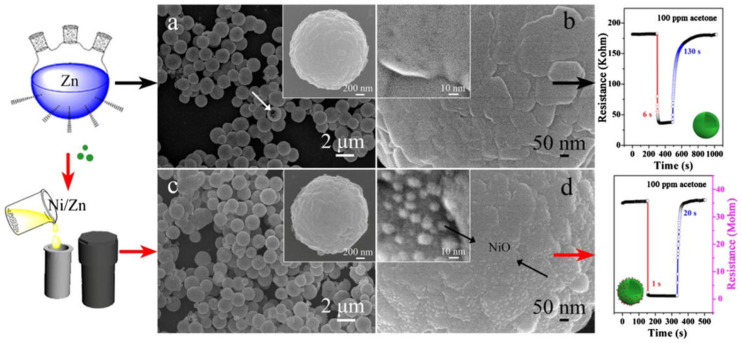
(**a**–**d**) Schematic representation of solvothermal synthesize, SEM images and sensory response of of NiO/ZnO hollow spheres towards acetone (Reproduced with the permission from Ref. [122]).

Towards acetone selective sensing, nanoflakes were incorporated in devices, which showed good sensitivity as other nanostructures. To this approach, α-MoO_3_ nanoflakes, SnS nanoflakes, and Au NPs incorporated MoS_2_ nanoflakes were developed by researchers by means of RF sputtering, solid-state reaction, and chemical exfoliation, respectively [117,118,119]. Wherein, SnS nanoflakes seems to be an impressive candidate with a sensor response of >1000 at low operating temperature (100 °C). Moreover, the sensor response is stable even after six weeks with a LOD of <5 ppm and response/recovery time of <15 s. Similar to the microspheres [120,121], materials with nanosphere (NSP) structures are also effectively applied in the acetone estimation as noted below. Liu et al. and Zhu et al. developed the NiO/ZnO and WO_3_-SnO_2_ hollow composite NSPs to engage in effective quantitation of acetone via solvothermal and hydrothermal methods, correspondingly [122,123]. As shown in Figure 7, solvothermally synthesized NiO/ZnO NSPs show great responses to acetone (R_a_/R_g_ = 29.8 for 100 ppm at 275 °C) with an LOD down to sub-ppm level [122].

In fact, the greater sensor response of NiO/ZnO NSPs was attributed to the decoration of NiO nanoparticles over the ZnO NSPs, thus become a notable candidate in acetone detection. On the other hand, the WO_3_-SnO_2_ forms two kind of NSPs, namely, the WO_3_-SnO_2_ 160 NSPs and the WO_3_-SnO_2_ 190 NSPs (prepared by heating at 160 °C and 190 °C, respectively) which display good response to acetone (R_a_/R_g_ = ~8 & 16 for 50 ppm at 275 °C). However, it still requirse further optimization to attain high linear responses and low LOD.

To this track, researchers described the acetone quantitation by flower-like structures of Na-doped p-type ZnO, cubic-rhombohedral-In_2_O_3_, Au NPs functionalized ZnO, and RuO_2_ modified ZnO [124,125,126,127]. In particular, the Na-doped ZnO nanoflowers revealed the acetone sensing under ultraviolet (UV) illumination with a LOD of 0.2 ppm [124]. Contrast to the cubic-rhombohedral-In_2_O_3_ microflowers (R_a_/R_g_ = 13.6 for 50 ppm at 250 °C; response/recovery time = 2 s/46 s; LOD = 0.01 ppm) and RuO_2_ modified ZnO nanoflowers (R_a_/R_g_ = 125.9 for 100 ppm at 172 °C; response/recovery time = 1 s/52 s; LOD ≤ 25 ppm) [125,127], Wang et al. described an exceptional sensor response of Au NPs functionalized ZnO nanoflowers [126] as shown in Table 1. Due to the loading of Au NPs over ZnO surface, the response is 2900 for 100 ppm acetone with a LOD of <20 ppm at higher operating temperature (~365 °C), thereby become a notable candidate in acetone quantification. However, the working temperature requires further optimization.

Majority of nanostructures showed the importance of the pore effect on acetone detection as described in the following. Porous nanoparticles (Au sensitized Fe_2_O_3_ NPs and Au/ZnO NPs), porous nanorods (ZnFe_2_O_4_ NRds and α-Fe_2_O_3_/SnO_2_ NRds), porous nanospheres (Pt sensitized W_18_O_49_), nanoporous fibers (ZnO/C), porous hierarchical nanostructures (Pt doped 3D SnO_2_ and Ni doped ZnO), and porous nanocomposites (CuFe_2_O_4_/α-Fe_2_O_3_ and (WO_3_/Au) were utilized by researchers towards acetone quantification [127,128,129,130,131,132,133,134,135,136,137]. These nanostructures were synthesized through various hydrothermal/atomic layer deposition/template mediated tactics. As shown in Table 1, many of these porous structures revealed exceptional acetone sensing due to the factor of pore effect in the gaseous species adsorption. In particular, Pt-sensitized W_18_O_49_ nanospheres and Pt-doped 3D porous SnO_2_ hierarchical structures [132,134] reported exceptional sensing of acetone (R_a_/R_g_ = 85 (for 20 ppm) and 505.7 (for 100 ppm) at 180 °C and 153 °C, respectively) with the LODs down to sub-ppm level (~0.05 ppm), thereby become the unique candidates. Owing to the porous effect on VOCs detection, ZnO/C nanoporous fibers [133] evidenced the sensitivity to both acetone and ethanol (R_a_/R_g_ = 53.222 and 59.273 (for 100 ppm) at 370 °C) with LODs down to sub-ppm level. But further optimizations are required on the operating temperature and selectivity to particular VOC.

**Figure 8:**
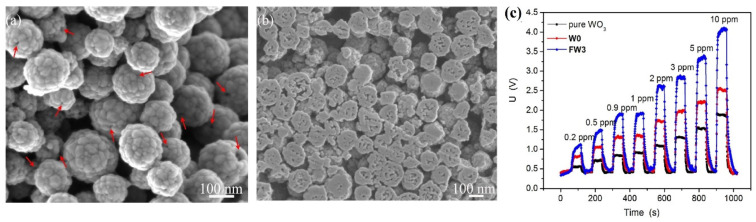
(**a**,**b**) Top-view and cross-sectional SEM image of Fe and C codoped WO_3_ (red arrow signifying the walnut like architecture); (**c**) Dynamic response-recovery curves of the three sensors based on the prepared pure WO_3_, C codoped WO_3_ (W0) and Fe and C codoped WO_3_ (FW3) sensors to different concentrations of acetone at 300 °C (Reproduced with the permission from Ref. [145]).

Other than porous nanostructures, hierarchical nanostructures synthesized by either hydrothermal or thermal oxidation tactics were effectively applied in acetone detection. Hierarchical nanostructures of ZnO NWs-loaded Sb-doped SnO_2_-ZnO, 3D flower-like ZnO, and Au NPs-SnO_2_ NTs seems to be impressive in the selective detection of acetone at operating temperatures ≥ 200 °C as presented in Table 1 [138,139,140]. Wherein, Wang et al. described the direct transformation of SnS_2_ NShs to hierarchical porous SnO_2_ NTs via thermal oxidation, which evinced a better selectivity upon Au NPs decoration at 200 °C with a LOD of 0.445 ppm, thereby stated as an exceptional candidate in acetone sensing [140]. To this light, differently shaped nanostructures were synthesized by the tactics like hydrothermal, impregnation, templated synthesize, etc. 3D inverse opal (3DIO) In_2_O_3_–CuO nano-architecture, 3D grass-like carbon-doped ZrO_2_ nano-architecture, Sm_2_O_3_ loaded mulberry shaped SnO_2_ hierarchical nanostructure, cactus-like WO_3_-SnO_2_ nanocomposite, walnut like architecture of Fe and C co-doped WO_3_, Urchin like Cr-doped WO_3_ hollow nanospheres, and core-shell heterostructure of ZnO/MoS_2_ NShs were engaged in effective detection of acetone as shown in Table 1 [141,142,143,144,145,146,147]. Wherein, 3D grass-like carbon-doped ZrO_2_ architecture film displays the selectivity to both alcohols and acetone [142], thereby cannot be stated as a better candidate for acetone detection. Similarly, as seen in Figure 8, walnut like architecture of Fe and C co-doped WO_3_, lying underneath microstructure rather than nanostructure, shows high selectivity to acetone (R_a_/R_g_ = ~18 for 10 ppm) at 300 °C even after 12 weeks [145]. Moreover, it can detect the acetone even at sub-ppm concentration (~0.2 ppm) and the sensor can operates at 90% humid condition, thus become an exceptional candidate in acetone quantitation. Apart from the aforementioned diverse nanostructures, nanocomposites that are not described under any unique nano-architectures also show their high selective sensitivity to acetone [148,149,150,151,152,153] as shown in Table 1. Wherein, a few of them operate at diverse humid conditions (10–90% RH) and some others require optimization of operating temperature to attain high sensitivity.

## 4. Alcoholic Vapor Detection by Miscellaneous Nanostructures

In regard to the development of alcoholic vapor detection, various nanostructured materials were placed in the gaseous chamber to interact with the adsorbed oxygen species follow the reactions described in Equations (3) and (4) to release electrons.

(C_n_H_2n+1_ OH)_gas_ → (C_n_H_2n+1_ OH)_ads_ (3)

(C_n_H_2n+1_ OH)_ads_ + 3nO^−^ → nCO_2_ + (n+1)H_2_O + 3ne^−^ (4)

Similar to the above detection process, metal oxide can oxidize the alcoholic vapor to aldehyde and convert them to water and carbon-dioxide to produce electrons as described in Equations (5)–(7).

(C_n_H_2n+1_ OH)_ads_ → (C_n_H_2n+1_ O^−^)_ads_ + H^+^ (5)

(C_n_H_2n+1_ O^-^)_ads_ + H^+^ → (C_n_H_2n+1_ O)_ads_ + H_2_ (6)

(C_n_H_2n+1_ O)_ads_ + (3n − 1)O^-^_ads_ → nCO_2_ + (n + 1)H_2_O + 3(n−1)e^−^ (7)

In the above two sensory processes, the released electrons lead to changes in resistance, which is then used as a sensor signal (R_a_/R_g_). To this track, numerous reports with diverse nanostructures have been reported towards the sensing of alcoholic gases as detailed as following.

Through the synthetic tactics like soft-chemical approach, hydrothermal, calcination, solvothermal, and sol–gel methods, researchers proposed several nanoparticles syntheses and applications in the alcoholic vapors sensing [154,155,156,157,158]. Sn_3_N_4_ NPs, Ni-doped SnO_2_ NPs, C-doped TiO_2_ NPs, Pr-doped In_2_O_3_ NPs, and Au/Cl co-modified LaFeO_3_ NPs were engaged in the detection of alcoholic vapors. In particular, Sn_3_N_4_ NPs and Au and Cl co-modified LaFeO_3_ NPs [154,158] displayed good sensor responses to ethanol vapor with decent response/recovery time at an optimum operating temperature of 120 °C as shown in Table 2. Whereas, C-doped TiO_2_ NPs were demonstrated in the detection of n-pentanol at 170 °C with a LOD down to sub-ppm level [156]. In this light, Ni-doped SnO_2_ NPs were noted as an exceptional candidate, which showed sensitivity to both n-butanol and formaldehyde via tuning the doping concentrations of Ni ions [155]. Two percent Ni-doped SnO_2_ NPs displayed a remarkable sensor response (R_a_/R_g_ = 1690.7) to n-butanol at 160 °C with a response/recovery time of 10 s/>10 min. Moreover, 4% Ni-doped SnO_2_ NPs demonstrated a sensor response (R_a_/R_g_ = 1298) at 100 °C with the response/recovery time of 6 s/>10 min. Their detection of n-butanol displayed a linear response from 1 to 100 ppm with a LOD of ~1 ppm, thereby can be considered for future development.

Similar to the NPs, nanocrystalline materials were also used in the sensing applications of alcohols. Cao and co-workers explored the ethanol sensing utilities of unmodified/Cl-modified LaFe_x_O_3-δ_ nanocrystals [159,160]. Both LaFe_x_O_3-δ_ and Cl-modified LaFe_x_O_3-δ_ nanocrystals displayed sensor responses of 132 and 79.2 (for 1000 and 200 ppm, correspondingly) with the response/recovery time of <10 s at 140 °C and 136 °C, respectively. However, the proposed LODs of both nanocrystals require further optimization. Ethanol sensing was also delivered by α-MoO_3_ and copper oxide (CuO/Cu_2_O) nanocrystals [161,162], but their operating temperatures seems to be >250 °C (see Table 2), thus requires more interrogations. Xiaofeng et al. described the methanol detection by Gd_1–x_Ca_x_FeO_3_ (x = 0–0.4) nanocrystalline powder [163]. This material showed a sensor response of R_a_/R_g_ = 117.7 (for 600 ppm) at 260 °C with a response/recovery time of <2 min and a LOD of <50 ppm. However, it still requires investigations on more interfering gaseous.

**Figure 9:**
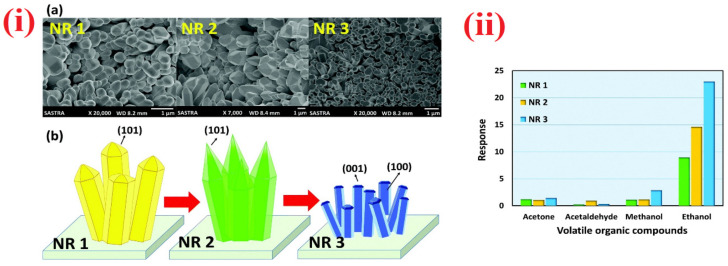
(**i**) (**a**) FESEM images of PVA–ZnO nanocomposites calcined at 873 K for different time durations—1 h (NR1), 3 h (NR2), and 6 h (NR3) and (**b**) schematic of the ZnO nanorods (NR1, NR2, and NR3); (**ii**) Sensing responses of the calcined samples towards 100 ppm of acetone, acetaldehyde, methanol, and ethanol at room temperature (299 K) (Reproduced with the permission from Ref. [169]).

Towards the detection of ethanol, doped/functionalized nanowires, such as Au modified ZnO NWs, Fe_2_O_3_ NPs coated SnO_2_ NWs, In_2_O_3_ NPs decorated ZnS NWs, and Sr-doped cubic In_2_O_3_/rhombohedral In_2_O_3_ homojunction, NWs displayed extensive sensitivity [164,165,166,167]. These NWs were synthesized by vapor–liquid–solid (VLS) method, hydrothermal or electrospun tactics. All NWs can operate at 300 °C except the Au modified ZnO NWs, which operate at 350 °C. In particular, Sr-doped cubic In_2_O_3_/rhombohedral In_2_O_3_ homojunction NWs [167] are impressive in terms of the response/recovery time (<1 min) with sub-ppm LOD (0.025 ppm). Similar to the NWs, nanorods are also utilized in the quantitation of ethanol as follows. Cr_2_O_3_ NPs functionalized WO_3_ NRds, ZnO NRds, Pd NPs decorated ZnO NRds, and SnO_2_-ZnO heterostructure NRds were synthesized by thermal evaporation, hydrothermal, and chemical vapor deposition (CVD) methods and employed in ethanol sensing [168,169,170,171,172]. Shankar et al. reported the fabrication of three kinds of polyvinyl alcohol (PVA)-ZnO NRds calcined composites (NR1, NR2, and NR3) and demonstrated their exceptional sensitivity to ethanol at room temperature as shown in Figure 9 and Table 2 [169]. In this work, NR3 displays a good sensing performance (R_a_/R_g_ = 23; response/recovery time =26 s/43 s; LOD ≤ 5 ppm). In view of this, SnO_2_-ZnO heterostructure NRds (operating temperature is 275 °C) seems to be another good candidate with the LOD of 1 ppm, but it still requires optimization to reduce the operation temperature.

On the other end of the spectrum, Perfecto et al. reported the discrimination of Iso-propyl alcohol (IPA) by rGO-WO_3_.0.33H_2_O nanoneedles (rGO: reduced graphene oxide) [173]. This material was synthesized by the combination of ultrasonic spray nozzle (USN) and microwave-assisted hydrothermal (MAH) methods and displayed a sensor response of 4.96 to 100 ppm IPA at room temperature and at 55% RH with a LOD of 1 ppm. Therefore, it becomes an impressive candidate in IPA sensing. However, more investigations are required to improve the sensitivity. Sm-doped SnO_2_ nanoarrays were developed through a hydrothermal tactic and applied in IPA sensing by Zhao and co-workers [174]. In which, it showed a high sensing response to IPA (R_a_/R_g_ = 117.7; response/recovery time = 12 s/20 s; at 252 °C) with a LOD of ~ 1 ppm, therby become a notable material. However, this work requires more efforts in the reduction of operating temperature.

With regard to different alcoholic vapors detections, materials with nanofibers structures were proposed by many research groups. For example, Han et al. developed the rough SmFeO_3_ nanofibers via electrospinning and calcination processes and used in the determination of ethylene glycol [175]. The sensor response reached 18.19 to 100 ppm of ethylene glycol at 240 °C with a LOD of ~5 ppm. Moreover, both response/recovery time were less than a min, thus become a prominent material in ethylene glycol sensor. Similar to the above report, Feng and co-workers synthesized the In-doped NiO nanofibers through electrospinning method and employed in the detection of methanol [176]. At 300 °C, the sensor response of In-doped NiO nanofibers reached 10.9 for 200 ppm methanol (response/recovery time = 273 s/26 s), which was five times higher than that of pure NiO nanofibers. However, this work needs further optimization to minimize the working temperature and LOD (25 ppm). In view of this, nanofibrous materials (SiO_2_@SnO_2_ core-shell nanofibers and Yb doped In_2_O_3_ nanofibers) were also engaged in the sensing of ethanol [177,178]. SiO_2_@SnO_2_ core-shell nanofibers [177] were synthesized by electrospinning and calcination process, but details on the sensor studies seems to be deficient (see Table 2) and need more interrogations. In contrast, Yb-doped In_2_O_3_ nanofibers (developed by electrospinning) is an impressive candidate with the capability of operation at room temperature and a sensor response of 40 for 10 ppm ethanol with a LOD of 1 ppm [178].

**Figure 10:**
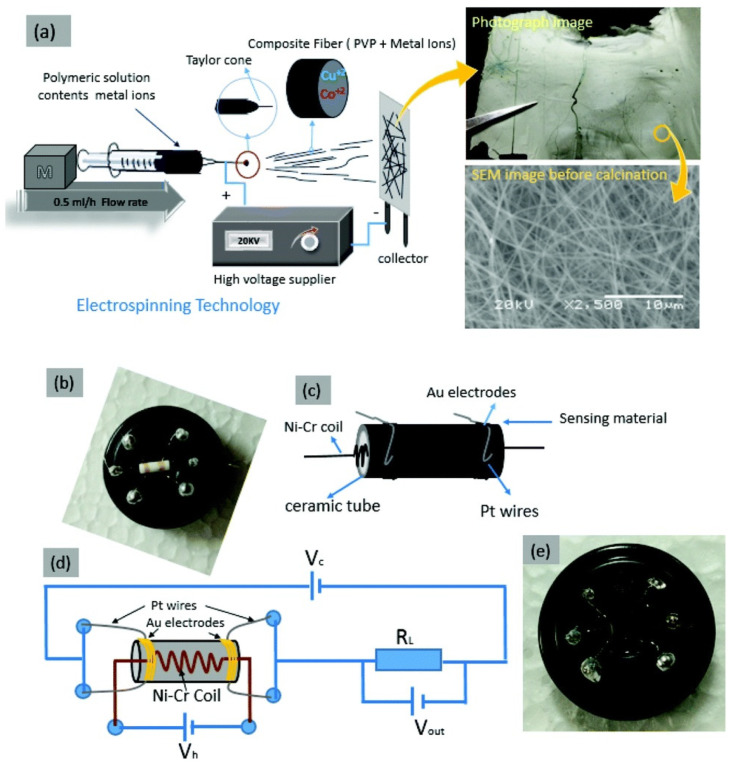
(**a**) A diagram of the electrospinning process with a photograph of as-spun fiber mat and SEM image of composite fibers before calcination treatment. (**b**,**e**) Photographs of the sensor without and with covering by sensing materials, respectively. (**c**) Schematic diagram of the sensor and its components. (**d**) A schematic of the sensor circuit and its elements (Reproduced with the permission from Ref. [179]).

Next, nanotubes were employed in the alcoholic vapor detection by using changes in resistance as the sensor signal. For example, Alali and co-workers developed the p-p heterojunction CuO/CuCo_2_O_4_ NTs through electrospinning and applied in the sensing of n-propanol at room temperature as shown in Figure 10 [179]. The importance of this work lies on its sensor signal to 10 ppm of n-propanol (R_a_/R_g_ = 14; response/recovery time = 6.3 s/4.1 s). Similar to the above work, p-p type CuO–NiO heterojunction NTs were synthesized via calcination treatment and utilized in the detection of glycol at 110 °C [180]. Herein, the sensor responses reached 10.35 for 100 ppm glycol with a response/recovery time of 15 s/45 s and a LOD down to sub-ppm level (0.078 ppm), thus become a remarkable material. Ethanol sensing was demonstrated by coated and doped materials with nanotubes structures (NiO decorated SnO_2_ NTs, Ca-doped In_2_O_3_ NTs, Ni-doped In_2_O_3_ NTs, and W-doped NiO NTs) at diverse operating temperatures [181,182,183,184]. These nanotubes were synthesized by hydrothermal or electrospinning tactics and successfully used in the discrimination of ethanol. The NiO decorated SnO_2_ NTs [181] showed ethanol sensing ability of vertical standing NTs at 250 °C, but was low in sensitivity and LOD (R_a_/R_g_ = 123.7 for 1000 ppm; response/recovery time = 10 s/58 s). On the other hand, enhanced sensor responses were observed in the rest of the doped NTs at ≥ 160 °C with a LODs of ~5 ppm. For example, Ca-doped In_2_O_3_ NTs [182] reached a highest sensor response of 183.3 for 100 ppm ethanol (response/recovery time = 2 s/56 s) with a LOD of <5 ppm at 240 °C, thus, is noted as a good material for ethanol sensors.

Discrimination of ethanol vapor was demonstrated by materials with nanobelt structures as discussed in the following. In_2_O_3_ NPs deposited TiO_2_ nanobelts, α-MoO_3_ nanobelts, and Zn-doped MoO_3_ nanobelts were hydrothermally prepared and engaged in ethanol detection at 100 °C, 300 °C, and 240 °C, respectively [185,186,187]. Among them, In_2_O_3_ NPs deposited TiO_2_ nanobelts [185] seems to be an impressive candidate with a sensor response of >9 for 100 ppm ethanol, working temperature at 100 °C, response/recovery time of 6 s/3 s), and a LOD of 1 ppm as noted in Table 2. To this track, Wang et al. described the n-butanol sensing utility of nanocube structured Fe_2_O_3_ that was derived from MOF via calcination [188]. Upon the exposure to 100 ppm n-buatnol, the MOF derived Fe_2_O_3_ nanocubes displayed a sensor response of ~6 (for 100 ppm) at 160–230 °C, response/recovery time is <2 min with a LOD of <1 ppm. Similarly, Nguyen et al. delivered the ethanol sensor property of hydrothermally synthesized In_2_O_3_ nanocubes [189]. In which, the sensor response reached 85 at 300 °C for 100 ppm ethanol with lower response/recovery time (15 s/60 s) and a LOD of <5 ppm, thus is noted as a noteworthy material in ethanol detection. However, further optimization is required in reducing the working temperature.

Other than the microcages [190], nanocages were also reported in ethanol quantitation by the researchers. ZIF-8 derived ZnO hollow nanocages, ZIF-8 derived-Ag-functionalized ZnO hollow nanocages, and Cu_2_O hollow dodecahedral nanocages were employed in the exceptional detection of ethanol [191,192,193]. Among them, both ZIF-8 derived ZnO hollow nanocages and ZIF-8 derived-Ag-functionalized ZnO hollow nanocages were reported with high sensor responses to 100 ppm ethanol (R_a_/R_g_ = 139.41; response/recovery time = 2.8 s/56.4 s at 325 °C and R_a_/R_g_ = 84.6; response/recovery time = 5 s/10 s at 275 °C, correspondingly) with their LODs down to sub-ppm levels (0.025 and 0.0231 ppm, respectively). In the light of this, such materials developments are appreciated with further interrogations to reduce the working temperature. Nanosheet structured materials were also developed for the quantification of gaseous ethanol. Wherein, Al-doped ultrathin ZnO NShs, NiO NPs decorated SnO_2_ NShs, and CuO NPs decorated ultrathin ZnO NShs displayed their sensor responses to ethanol vapor [194,195,196]. However, apart from sensor responses and LODs, these materials require optimization for working temperatures, which are >250 °C, as noted in Table 2.

**Figure 11:**
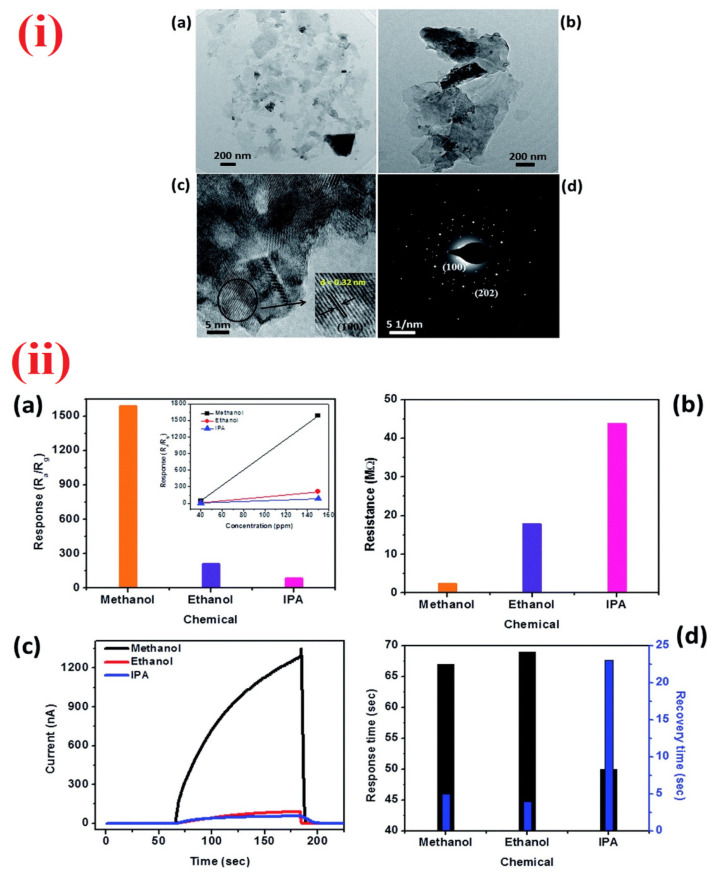
(**i**) TEM analysis of SnS_2_ nanoflakes (**a** and **b**) low magnification TEM images, (**c**) high resolution TEM image and (**d**) selected area diffraction (SAED) pattern; (**ii**) Alcohol sensing performance of SnS_2_ nanoflakes (all alcohols at 150 ppm) at 25 °C (**a**) response vs. alcohols and inset shows the response vs. concentration, (**b**) resistance vs. alcohols, (**c**) typical *I–t* plot and (**d**) bar diagram of response and recovery time of the test alcohols (Reproduced with the permission from Ref. [197]).

In addition to diverse nanostructure-based alcoholic vapor detection, SnS_2_ and CdS nanoflakes were proposed for the sensing of methanol and IPA [197,198]. Bharatula and co-workers identified the methanol sensing performance of SnS_2_ nanoflakes [197]. As illustrated in Figure 11, the SnS_2_ nanoflakes exhibit an exceptional sensor response of 1580 for 150 ppm gas exposure at room temperature with the response/recovery time of 67 s/5 s, thereby can be commercialized towards the detection of methanol. However, more investigations on interference studies are required. In view of this, Liu et al. demonstrated the IPA sensing properties of CdS nanoflakes [198] with a LOD down to sub-ppm level (0.05 ppm). However, the sensor studies of CdS nanoflakes require high temperature (275 °C) and also lack interference studies.

**Figure 12:**
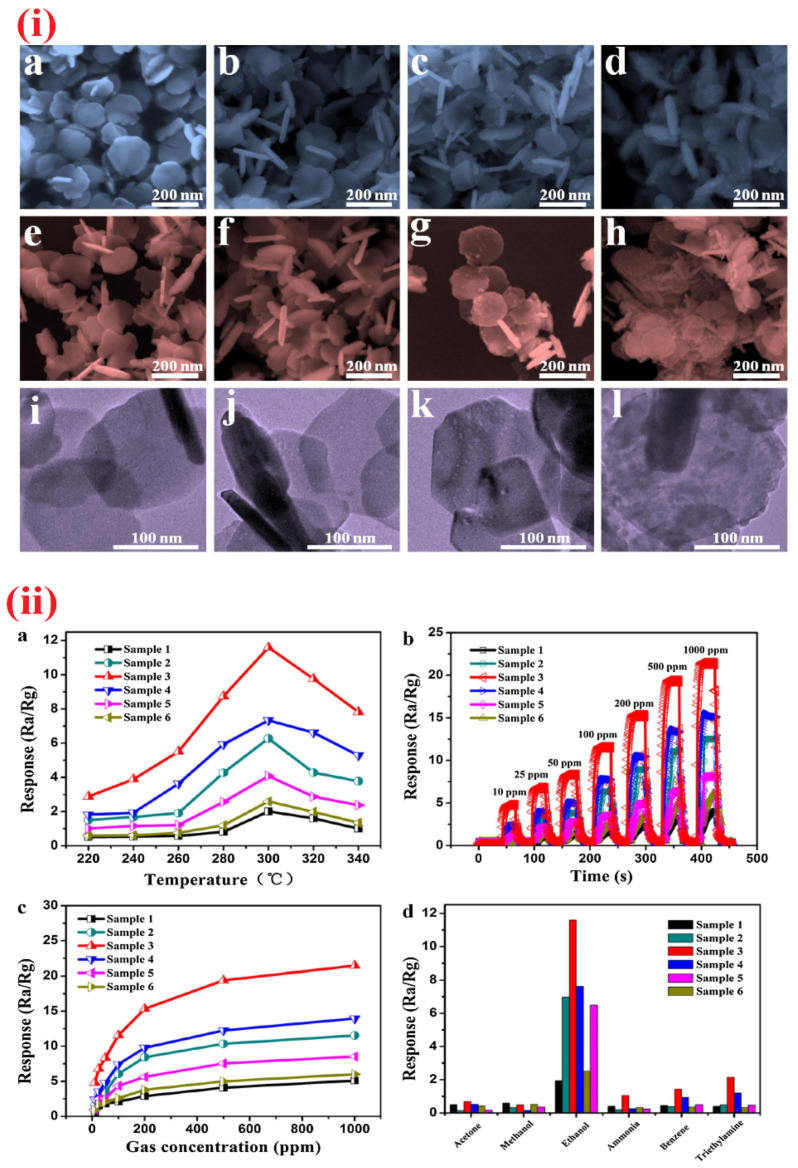
(**i**) SEM images of Sample 1–4: (**a**–**d**) before calcination; (**e**–**h**) after calcination; TEM images of Sample 1–4 after calcination (**i**–**l**); (**ii**) (**a**) The response of samples to 100 ppm ethanol at different operating temperature; (**b**) the response and recovery curves of samples upon exposure to 10–1000 ppm ethanol; (**c**) the response curves of samples to ethanol concentrations; (**d**) the linear relationship of log(S-1)-log(C) plot to ethanol at the optimum operating temperatures (Reproduced with the permission from Ref. [200]).

Materials such as Co-doped ZnO hexagonal nanoplates, ZIF-8 derived α-Fe_2_O_3/_ZnO/Au hexagonal nanoplates, and ZnO nanoplates were reported in the ethanol detection [199,200,201]. The sensor responses of those nanoplates were found as 570, 170, and 8.5 for 300, 100, and 1000 ppm of ethanol at 300 °C, 280 °C, and 164 °C, correspondingly. Wherein, hydrothermally synthesized Co-doped ZnO hexagonal nanoplates displayed a high sensor response to both ethanol and acetone [199]. However, further works on the interference studies as well as optimization for working temperature are required. On the other hand, compared to ZnO nanoplates [201], samples of ZIF-8 derived α-Fe_2_O_3/_ZnO/Au hexagonal nanoplates (synthesized by multi-step reaction process) [200] seems to be impressive in terms of response/recovery time (5 s/4 s) and LOD (~10 ppm) as shown in Figure 12. Materials with NSPs structures towards alcoholic gas detection were proposed by the researchers.

Nanosphere-shaped materials such as Zn_2_SnO_4_, monodispersed indium tungsten oxide, Ag@In_2_O_3_, ZnSnO_3_, ZnO, and α-Fe_2_O_3_ were effectively applied in the discrimination of alcoholic vapors as presented in Table 2 [202,203,204,205,206,207]. Wherein, Zn_2_SnO_4_ NSPs, and Ag@In_2_O_3_ core-shell NSPs [202,204] were found to be effective in detecting the ethanol with sensor response of 23.4 and 72.56 (for 50 ppm ethanol at 180 °C and 220 °C, individually) with response/recovery time of <1 min and LODs of ~5 ppm and ~2 ppm, correspondingly. Similarly, monodispersed indium tungsten oxide ellipsoidal NSPs and α-Fe_2_O_3_ hollow NSPs [203,207] were engaged in the quantitation of methanol at higher working temperature (>250 °C). However, α-Fe_2_O_3_ hollow NSPs were found to be more impressive with a sensor response of 25 for 10 ppm methanol at 280 °C (response/recovery time = 8 s/9 s) and with a LOD of 1 ppm. Subsequently, perovskite type ZnSnO_3_ NSPs and ZnO hollow NSPs [205,206] were applied in the detection of n-propanol and n-butanol (for 500 ppm at 200 °C and 385 °C, respectively). Between them, ZnSnO_3_ NSPs seems to be a better candidate with respect to their working temperature and LOD (0.5 ppm).

**Figure 13:**
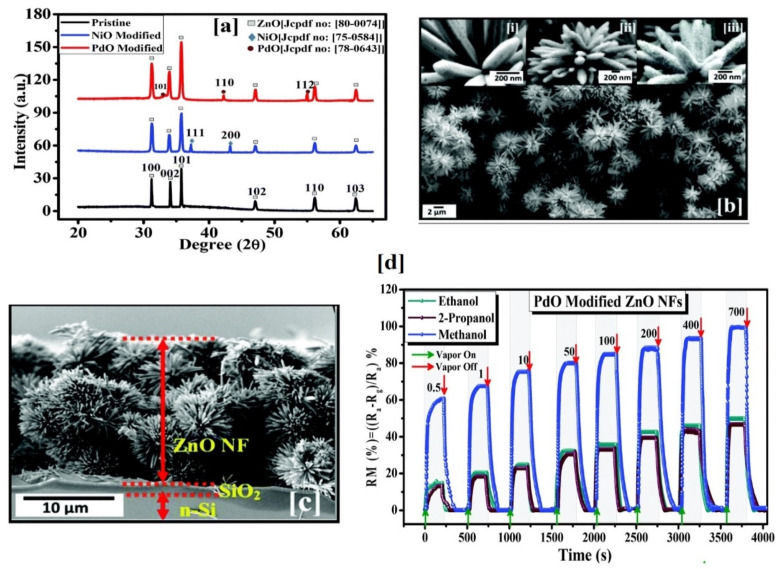
(**a**) X-ray diffraction patterns of the synthesized pristine ZnO NFs, NiO modified ZnO NFs, and PdO modified ZnO NFs. (**b**) FESEM image of pristine ZnO NF; inset shows magnified view of (**i**) pristine, (**ii**) NiO modified and (**iii**) PdO modified ZnO NF. (**c**) Cross-sectional view of grown ZnO NF on Si/SiO_2_ substrate. (**d**) Transient response characteristics (response magnitude (%) as a function of time) of the PdO–ZnO NF hybrid structure towards methanol, ethanol, and 2-propanol in the concentration range of 0.5–700 ppm at 150 °C (Reproduced with the permission from Ref. [208]).

In light of this, materials with modified nanoflower structures (PdO NPs modified ZnO, rGO nanosheets modified NiCo_2_S_4_ and Pd and rGO modified TiO_2_) and grained nanoflowers (NiO) were utilized in alcoholic gases assays [208,209,210,211]. PdO NPs modified ZnO nanoflowers displayed their enhanced sensing capability of methanol via decoration of PdO NPs over the surface of ZnO as shown in Figure 13. In a similar fashion, reduced graphene oxide (rGO) nanosheets modified NiCo_2_S_4_ nanoflowers and Pd and rGO modified TiO_2_ nanoflowers were demonstrated in the detection of ethanol as noted in Table 2.

In particular, rGO modified nanoflowers were highly impressive in terms of the operating temperature (≤100 °C) and can be commercialized in future. Nanomaterials with porosity plays a vital role in the sensing studies of volatile alcoholic compounds. Porous structures of Ag-functionalized ZnO, Al-doped ZnO, Au loaded WO_3_, 3D-ordered In-doped ZnO, Si@ZnO NPs, Ag loaded graphitic C_3_N_4_, hierarchical mixed Pd/SnO_2_, SnO_2_ fibers, hierarchical branched TiO_2_-SnO_2_, and hierarchical Co-doped ZnO were reported for their alcohol sensing utilities [212,213,214,215,216,217,218,219,220,221]. These porous nanostructures were synthesized by combustion method, nanocasting method, template mediated synthesis, microemulsion method, microdispensing method, solvothermal method, and calcination tactics. As noted in Table 2, these meso-/macro-porous nanostructures displays their good responses to alcoholic vapors at different working temperatures (lie between 150 and 350 °C). For example, hierarchical Co-doped ZnO mesoporous structure [221] revealed its exceptional sensitivity to ethanol (R_a_/R_g_ = 54 for 50 ppm at 180 °C; response/recovery time = 22 s/53 s) with a LOD of 0.0454 ppm, thereby can be attested as a good candidate for ethanol sensing studies.

Next, hierarchical nanostructures/nanocomposites were reported towards the detection of alcoholic gases. For instance, hierarchical Fe_2_O_3_ NRds on SnO_2_ NSPs nanocomposites and MoO_3_-mixed SnO_2_ hierarchical aerogel nanostructures were employed in the quantification of ethanol at 320 °C and 260 °C, respectively [222,223]. The sensors responses of these materials are 23.512 and 714, correspondingly, with response/recovery time <1 min/7 min, as shown in Table 2. In a similar trend, hierarchical In_2_O_3_ NPs decorated ZnO nanostructure [224] displayed discrimination of n-butanol (R_a_/R_g_ = 218.3 for 100 ppm at 260 °C; response/recovery time = 22 s/53 s) with a LOD down to sub-ppm level, thereby become a notable material. Apart from hierarchical nanostructures, diverse shaped nanostructures were also used in volatile alcohols identification. Honeycomb-like SnO_2_-Si-NPA nanostructure, rambutan-like SnO_2_ hierarchical nanostructure, ZnO nano-tetrapods, raspberry-like SnO_2_ hollow nanostructure, snowflake-like SnO_2_ hierarchical architecture, sea cucumber-like indium tungsten oxide, hollow Pentagonal-Cone-Structured SnO_2_ architecture, and neck-connected nanostructure film of ZIF-8 derived ZnO were proposed in the detection of alcohols as noted in Table 2 [225,226,227,228,229,230,231,232]. These materials were synthesized by solvothermal, hydrothermal, thermal-annealing, calcination, or CVD tactics. However, ZnO nano-tetrapods [227] can be ruled out due to their combined sensing applicability in hydrocarbon detection.

As described in Table 2, nanocomposite architectures were also demonstrated to be effective towards the quantification of alcohols. Wherein, nanocomposites of flower like LaMnO_3_@ZnO, SnO_2_-Pd-Pt-In_2_O_3_, RGO-SnO_2_ NPs, SnO_2_-V_2_O_5_, ZnO:Fe, g-C_3_N_4_-SnO_2_, and Co_3_O_4_ nanosheet array-3D carbon foam are more impressive towards alcohols sensing studies [233,234,235,236,237,238,239]. Among them, SnO_2_-V_2_O_5_ nanocomposite [236] was demonstrated with its sensitivity to ethanol (~66% for 160 ppm) through local grain-to-grain conductivity, but details on other sensor properties, such as response/recovery time and LOD were not available. Moreover, RGO-SnO_2_ NPs composite [235] seems to be significant in terms of its capable operation between 24 and 98% humid conditions. This may be due to the presence of reduced graphene oxide along with the SnO_2_ NPs. ZnO:Fe nanostructured film [237] morphology was improved by UV treatment, which further enhanced its sensor response. Similarly, upon modification of SnO_2_ by g-C_3_N_4_ NShs [238], the ethanol sensitivity was improved.

## 5. Various Nanostructures in Volatile Aldehyde Detection

In addition to acetone or alcoholic vapors detection, volatile organic aldehydes quantitation is also become essential and diverse nanostructured materials were reported in the aldehyde gases quantification. This section describes published reports in detail. Majority of the research papers generally focused on the assay of formaldehyde (HCHO) via the following reaction mechanism [240], which are also applicable for other aldehydes detection. As described in Equations (8) and (9), the adsorbed oxygen in the sensor chamber first reacts to form the acid followed by interaction with oxygen anion to release electrons, which can be adopted as the sensor signal.

HCHO + O^−^(ads) → HCOOH + e^−^ (8)

HCHO + 2O^−^ → CO_2_ + H_2_O + 2e^−^ (9)

Nanoparticles, such as In_2_O_3_ NPs, molecularly imprinted polymers (MIPs) NPs, amorphous Eu_0.9_Ni_0.1_B_6_ NPs, Ni-doped SnO_2_ NPs, and NiO granular NPs films were utilized to detect the formaldehyde at diverse operating temperatures with sub-ppm LODs, as noted in Table 3 [241,242,243,244,245]. Among them, molecularly imprinted polymers (MIPs) NPs discriminated the formaldehyde by means of quartz crystal microbalance (QCM) tactic with a LOD of 0.5 ppm, thus can be noted as an additional approach for the formaldehyde detection. Moreover, amorphous Eu_0.9_Ni_0.1_B_6_ NPs [243] were noted as an exceptional candidate in formaldehyde quantitation due do their sensor response (R_a_/R_g_ ≥ 7 for 20 ppm; response/recovery time ≤ 20 s (for both)) at room temperature. Therefore, commercialization of amorphous Eu_0.9_Ni_0.1_B_6_ NPs towards HCHO detection is desirable.

Regarding the HCHO quantification, researchers developed nanowires (SnO_2_ NWs, p-CuO/n-SnO_2_ core-shell NWs, ZnO meso-structured NWs and RGO coated Si NWs) and nanorods (Co doped In_2_O_3_ NRds and Ag-functionalized and Ni-doped In_2_O_3_ NRds) via VLS, atomic layer deposition, low-temperature chemical synthesis, metal-assisted chemical etching method (MACE), thermal annealing methods and applied them in effective sensing of HCHO gas [246,247,248,249,250,251]. As noted in Table 3, many of these materials display exceptional sensitivity to HCHO at different working temperatures. Note that ZnO meso-structured NWs [248] are capable of operating at room temperature and show a high sensor response of 1223% to 50 ppm HCHO with a LOD of 0.005 ppm under UV, thereby attest as a motivational research. In view of this, Zhang et al. reported the utility of Ag-LaFeO_3_ with spheres, fibers, and cages architectures with sensor responses of 16, 14, and 23 for 1 ppm HCHO at 82 °C, 110 °C, and 70 °C, correspondingly [252]. This work has driven the utilization of diverse nanostructures in VOCs determination as described below.

**Figure 14:**
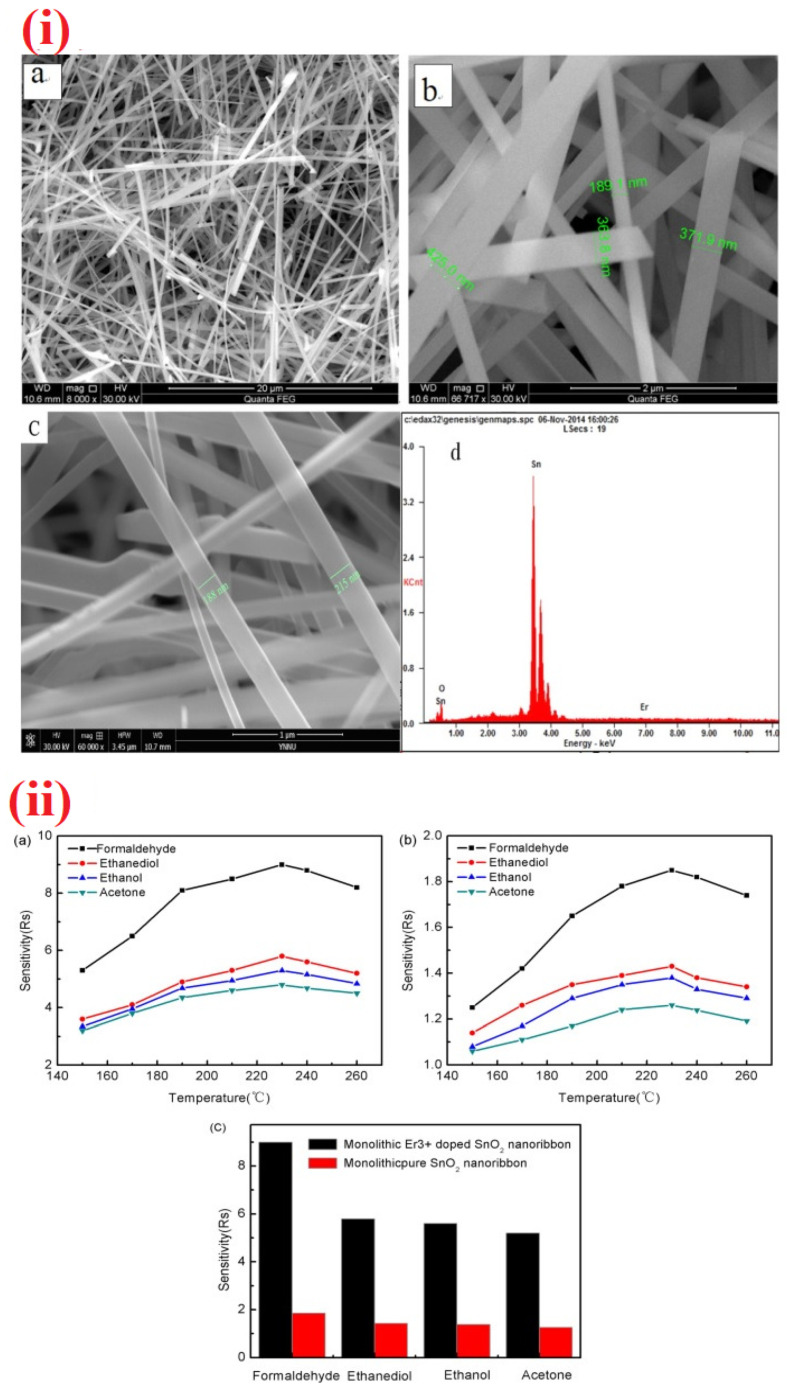
(**i**) SEM images of Er–SnO_2_ nanobelts (**a**) at a low magnification, (**b**) at a high magnification, (**c**) SEM image of pure SnO_2_ nanobelts, and (**d**) EDS spectra of Er–SnO_2_ nanobelts; (**ii**) (**a**) The responses (*R_s_*) versus temperature (*T*) of Er–SnO_2_ nanobelt to 100 ppm gas from 150 °C to 260 °C, (**b**) The responses (*R_s_*) versus temperature (*T*) of SnO_2_ nanobelt to 100 ppm gas from 150 °C to 260 °C, (**c**) Histogram of the sensitive responses at 230 °C (Reproduced with the permission from Ref. [258]).

Materials with nanofibers structures were also attested their sensing ability to HCHO vapor. Ag-doped LaFeO_3_ nanofibers, Co_3_O_4_-ZnO core-shell nanofibers, WO_3_/ZnWO_4_—1D nanofibers, and Pr-doped BiFeO_3_/hollow nanofibers were synthesized by electrospinning method with the combination of calcination tactic and utilized in HCHO detection at 190–230 °C [253,254,255,256]. As shown in Table 3, WO_3_/ZnWO_4_—1D hetrostructured nanofibers are found to be a good candidate in terms of the sensor response (R_a_/R_g_ = 44.5 for 5 ppm at 220 °C; response/recovery time = 12 s/14 s) with a LOD of 1 ppm [255]. However, further optimization to reduce the working temperature is still needed. Liang and co-workers presented the alkaline earth metals-doped In_2_O_3_ NTs for the sensing of HCHO [257]. In which, Ca-doped In_2_O_3_ NTs showed a sensor response of 116 for 100 ppm HCHO at 130 °C with the response/recovery time of 1 s/328 s and a LOD of 0.06 ppm. Therefore, it can be authorized as an exceptional material for the HCHO sensor studies. Doped nanobelts were employed in the discriminative assay of HCHO as detailed below. Er-doped SnO_2_ and Pt-decorated MoO_3_ nanobelts were reported for the quantitation of volatile HCHO [258,259]. As shown in Figure 14, Er-doped SnO_2_ nanobelts [258] display a distinct response to HCHO vapor (R_a_/R_g_ = 9 for 100 ppm at 230 °C; response/recovery time = 17 s/25 s) with a LOD of 0.141 ppm. On the other hand, Pt-decorated MoO_3_ nanobelts [259] are more impressive with a sensor response (R_a_/R_g_ = ~25% for 100 ppm; response/recovery time = 17.8 s/10.5 s; LOD = 1 ppm) at room temperature, thereby become a noteworthy material in HCHO sensory research.

**Figure 15:**
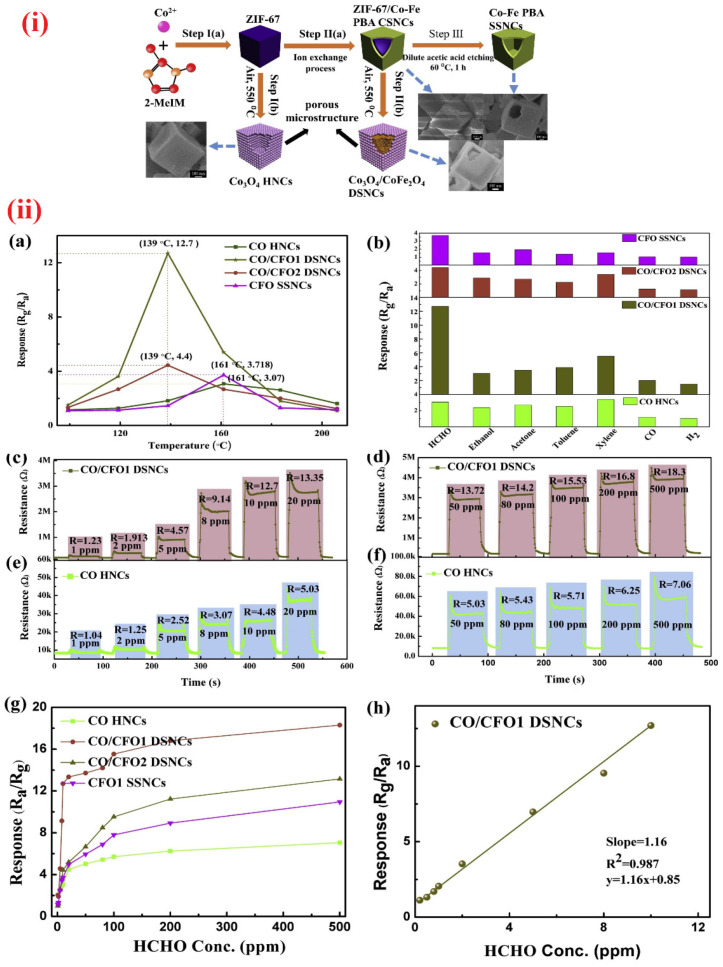
(**i**) Schematic of the process to fabricate porous Co_3_O_4_ HNCs, CCFO DSNCs, and CFO SSNCs; (**ii**) (**a**) Responses of the sensors to 10 ppm of formaldehyde at 80–230 °C. (**b**) Responses of the sensors to 10 ppm of various gases at the optimal operating temperatures. Dynamic response–recovery curves of the CCFO DSNCs and Co_3_O_4_ HNCs sensors to xylene in the ranges (**c**,**e**) 1–20 ppm and (**d**,**f**) 50–500 ppm under their optimal operating temperatures. (**g**) Response of the sensors in the ranges from 1 to 500 ppm of xylene. (**h**) Responses of the CCFO DSNCs sensor as a function of low formaldehyde concentration (1–10 ppm) (Reproduced with the permission from Ref. [262]).

Nanocube-shaped materials were consumed in the quantification of HCHO vapor at diverse operating temperatures. Multi-shelled hollow nanocubes (ZnSnO_3_ and ZnSn(OH)_6_; synthesized by co-precipitation method) were engaged in HCHO sensing at 220 °C and 60 °C, respectively [260,261]. The responses reached 37.2 and 56.6 (for 100 ppm; response/recovery time = 1 s/59 s and 1 s/89 s, respectively) with the LODs of <10 ppm and 1ppm, correspondingly. Both materials performed remarkablely in HCHO sensory studies. Similarly, MOF (zeolite imidazolate framework-67; ZIF-67)-derived Co_3_O_4_/CoFeO_4_ double-shelled nanocubes were utilized in the detection of HCHO [262]. As illustrated in Figure 15, the ZIF-67-derived Co_3_O_4_/CoFeO_4_ double-shelled nanocubes were synthesized by MOF route and found to be more effective in the sensing of HCHO even at 1 ppm concentration.

**Figure 16:**
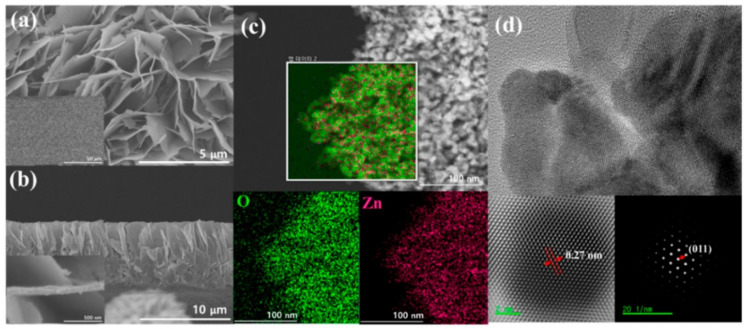
Field Emission Scanning Electron Microscopy (FESEM) image (**a**) cross section image (**b**), energy dispersive spectroscopy (EDS) mapping image (**c**) and transmission electron microscope (TEM) image (**d**) (inner HR TEM and selected area diffraction (SAED) pattern) of ZnO NShs (Reproduced with the permission from Ref. [265]).

For 10 ppm HCHO, the sensor response reaches 12.7 at low operating temperature (139 °C) with short response/recovery time (4 s/9 s) and sub-ppm LOD (0.3 ppm). Therefore, development of such nanocube materials are highly anticipated in HCHO quantitation. Nanosheets with and without decoration were effectively applied in the sensing of HCHO. High resolution SEM and TEM images of nanosheets are dispayed in Figure 16. WO_3_ clusters decorated In_2_O_3_ NShs (synthesized by impregnating method), SnO_2_ NShs, ZnO NShs, Au atom dispersed In_2_O_3_ NShs, and PdAu bimetal decorated SnO_2_ NShs were exploited in the discrimination of HCHO at various operating temperatures [263,264,265,266,267]. However, the ZnO NShs (adopted from hydrothermal method; Figure 16) were engaged in aqueous phase detection of HCHO with linear regression of 10 nM to 1 mM and a LOD of 210 nM; thus, it cannot be listed as device-based assays [265]. Compared to other HCHO sensory reports, Au atom dispersed In_2_O_3_ NShs (developed by light assisted reduction method) were highly fascinated with respect to its sensor reposes (R_a_/R_g_ = 85.67 for 50 ppm at 100 °C; response/recovery time = 25 s/198 s) with an exceptional LOD of 0.00142 ppm [266]. PdAu bimetal decorated SnO_2_ NShs (synthesized by hydro-solvothermal treatment) were reported for the detection of both acetone and HCHO at 250 °C and 110 °C, respectively, with a LODs down to sub-ppm level [267]. The device also works at high humid condition (94% RH), but interference studies still need more clarification., Hayashi et al. proposed the utilization of SnS_2_ nanoflake device to detect the HCHO gas [268], which had a LOD down to sub-ppm (0.001/0.02 ppm) and operated at 210 °C. However, details on other sensory properties are currently missing.

Nanospheres were also fabricated to quantify the HCHO as described in the following. Hussain and co-workers demonstrated the utilization of 0D ZnO NSPs and NPs (developed by low temperature hydrothermal route) to discriminate the formaldehyde [269]. Wherein, 0D ZnO NSPs displayed a higher sensor response (R_a_/R_g_ = 95.4 for 100 ppm at 295 °C; response/recovery time = 11 s/8 s; LOD as ~5 ppm) than that of 0D ZnO NPs (R_a_/R_g_ = 68.2 for 100 ppm at 295 °C; response/recovery time = 11 s/8 s; LOD = ~10 ppm). However, the working temperature needs to be reduced before commercialization. In light of this, hydrothermally synthesized Ag-doped Zn_2_SnO_4_/SnO_2_ hollow NSPs [270] were reported in the detection of HCHO at low working temperature (R_a_/R_g_ = 60 for 50 ppm at 140 °C; response/recovery time = 9 s/5 s; LOD = 5 ppm), thereby is attested as a better candidate. Similar to NSPs, microspheres were also still applied in the detection of HCHO. For example, MOF-derived ZnO/ZnCo_2_O_4_ microspheres [271] were used in HCHO sensing with a response of 26.9 for 100 ppm gas with response/recovery time of 9 s/14 s and a LOD of 0.2 ppm. Therefore, research on NSPs/microspheres-based VOCs detection is highly anticipated in future.

As an alternative to HCHO sensors, materials with nanoflowers like structures were developed. Hierarchical SnO_2_ and Sn_3_O_4_/rGO hetrostructured nanoflowers (synthesized by hydrothermal method) were reported for formaldehyde assay [272,273]. As noted in Table 3, Sn_3_O_4_/rGO hetrostructured nanoflowers are more effective in sensing HCHO (R_a_/R_g_ = 44 for 100 ppm at 150 °C; response/recovery time = 4 s/125 s) than that of Hierarchical SnO_2_ nanoflowers with a LOD of 1 ppm. Moreover, the presence of rGO enhances the sensor response and long-term stability, thereby is noted as a good addition to HCHO sensors.

Exploitation of different nanostructures with porosity in the quantitation of HCHO detection has been demonstrated. Porous nanostructures such as Au-loaded In_2_O_3_ hierarchical porous nanocubes, Ag-loaded ZnO porous hierarchical nanocomposite, Pd–WO_3_/m-CN mesoporous nanocubes, GO/SnO_2_-2D mesoporous nanosheets, ZnSnO3-2D mesoporous nanostructure, LaFeO_3_ porous hierarchical nanostructure, Bi doped Zn_2_SnO_4_/SnO_2_ porous nanospheres, ZnO porous nanoplates, and Au@ZnO mesoporous nanoflowers were reported as HCHO sensors at diverse operating temperatures as summarized in Table 3 [274,275,276,277,278,279,280,281,282]. The higher sensor responses achieved from those materials are attributed to the presence of porosity, which increases the adsorption of the HCHO gas significantly to enhance the signal (changes in resistance). Among the aforementioned porous materials, 2D mesoporous GO/SnO_2_ nanosheets [277] revealed an exceptional sensor response of 2275.7 at low operating temperature (60 °C) with the response/recovery time of 81.3 s/33.7 s and a LOD of 0.25 ppm. Therefore, this extraordinary work has demonstrated possible commercialization in future. Similarly, mesoporous Pd–WO_3_/m-CN nanocubes [276] performed quite impressively with operation at 95% dry humid condition. In the light of this, TiO_2_/ZnCo_2_O_4_ porous nanorods were engaged in the detection of HCHO and trimethylamine (TEA) at 220 °C and 130 °C [283]. However, response in TEA detection was higher than that of HCHO and it was lack of interference studies.

**Figure 17:**
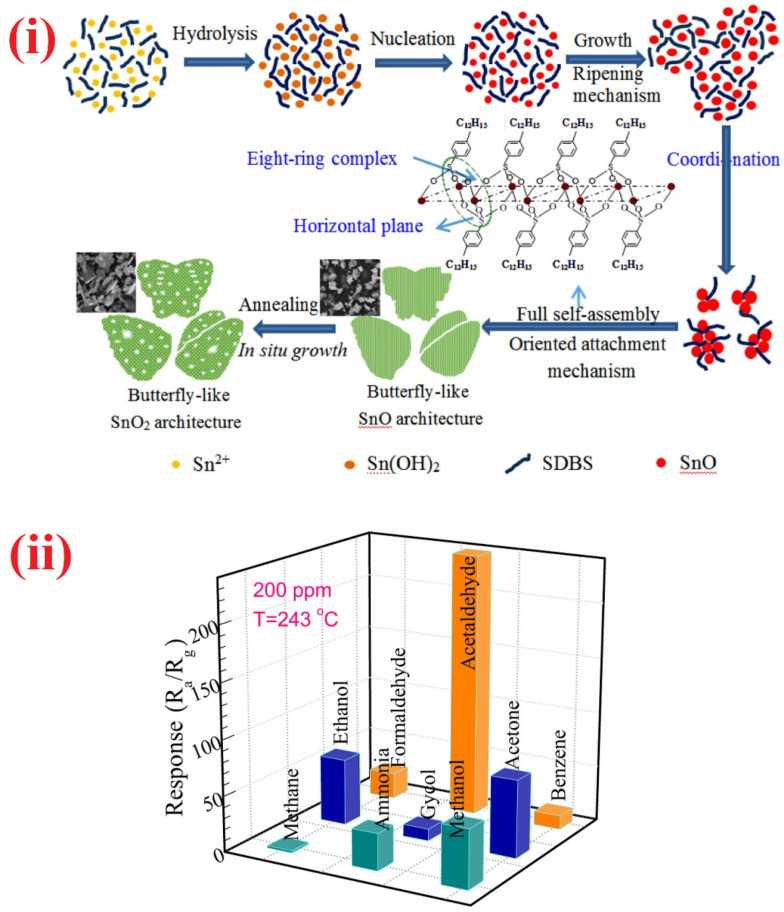
(**i**) Schematic illustration of the formation of butterfly-like SnO_2_ architectures. (**ii**) The response comparison of the butterfly-like SnO_2_ architectures to 200 ppm of various VOCs at the optimal operating temperature. (Reproduced with the permission from Ref. [290]).

Hierarchical nanostructures of Zn_2_SnO_4_/SnO_2_, Pt/MnO_2_-Ni(OH)_2_ hybrid nanoflakes, SnO_2_ nanofiber/nanosheets, In_2_O_3_@SnO_2_ composite, and cedar-like SnO_2_ were demonstrated as HCHO sensors [284,285,286,287,288]. These materials were synthesized by means of chemical route, hydrothermal, or electrospinning tactics. Wherein, hierarchical Pt/MnO_2_-Ni(OH)_2_ hybrid nanoflakes [285] demonstrated only the formaldehyde oxidation activity at room temperature, thereby more work was needed to study the exact sensor response. Among these hierarchical nanostructures, In_2_O_3_@SnO_2_ hierarchical composite displayed a good sensor response (R_a_/R_g_ = 180.1 for 100 ppm at 120 °C; response/recovery time = 3 s/3.6 s) with a LOD of 0.1 ppm. Different shaped nanostructures were also proposed by researchers as an alternative to aldehyde sensors. Following the similar synthetic approaches, urchin-like In_2_O_3_ hollow nanostructure, butterfly-like SnO_2_ hierarchical nanostructure, SnO_2_ hollow hexagonal prisms, and NiO/NiFe_2_O_4_ composite nanotetrahedrons were synthesized and used in aldehydes detection as illustrated in Table 3 [289,290,291,292]. However, butterfly-like SnO_2_ hierarchical nanostructure [290] performs better in the detection of acetaldehyde than that of HCHO as noted in Figure 17. It displays a high sensor response to acetaldehyde (R_a_/R_g_ = 178.3 for 100 ppm at 243 °C; response/recovery time = 28 s/58 s) with a LOD of <0.5 ppm, thereby cab be noted as an exceptional candidate. In the detection of HCHO, nanocomposite materials were also effectively employed. Vertical graphene (VG) decorated SnO_2_, multiwalled carbon nanotubes-polyethyleneimine, and n-n TiO_2_@SnO_2_ nanocomposites (synthesized by ALD method) were utilized in the discrimination of HCHO with LODs down to sub-ppm level [293,294,295]. In particular, multiwalled carbon nanotubes-polyethyleneimine composites [286] operated at room temperature with good response/recovery time (<1 min for both). On the other hand, n-n TiO_2_@SnO_2_ nanocomposites [295] detected the HCHO under UV and dark conditions at 50 °C. Therefore, development of such nanocomposite-based VOC sensor devices are much anticipated.

## 6. Various Nanostructures in Volatile Organic Amines Detection

The well-known toxic volatile organic amines detection by nanomaterials are highly anticipated. The mechanism follows the similar trend as proposed in other VOCs sensors. Upon interaction with anionic oxide species in the chamber, the volatile amines reacts and releases electrons resulting changes in resistance, which is adopted as sensor signals. A simple scheme represents the reaction mechanisms in triethylamine (TEA) detection is given in Equations (10)–(12). Similar to this mechanism, other volatile organic amine detections also follow the same sequence.

In chamber O_2_ (ads) + e^−^ → O_2_^−^ (ads) (10)

In chamber O_2_^−^ (ads) + e^−^ → 2O^−^ (ads) (11)

Under TEA (C_2_H_5_)_3_N + O^−^ (ads) → N_2_ + CO_2_ + H_2_O + e^−^ (12)

TEA detection was demonstrated by nanoparticle-based devices as explained below. Co_3_O_4_/ZnO hybrid NPs, Ho-doped SnO_2_ NPs, and CuCrO_2_ NPs (synthesized by hydrothermal or gas–liquid phase chemical method and annealing) were utilized in the sensing of TEA at 285 °C, 175 °C, and 140 °C, respectively, as shown in Table 4 [296,297,298]. Wherein, Co_3_O_4_/ZnO hybrid NPs [296] shows a high response (R_a_/R_g_ = 282.3 for 200 ppm at 285 °C; response/recovery time = 25 s/36 s) with a LOD of ~10 ppm. However, more investigations are needed to lower the working temperature. Subsequently, multi-metal functionalized tungsten oxide NWs (Ag/Pt/W_18_O_49_ NWs) and 1D SnO_2_ coated ZnO hybrid NWs were reported in the discrimination of TEA and n-Butylamine, correspondingly, at 240 °C. The Ag/Pt/W_18_O_49_ NWs [299] displayed a high sensor response to TEA (R_a_/R_g_ = 813 for 50 ppm at 240 °C; response/recovery time = 15 s/35 s (for 2 ppm) with a LOD of 0.071 ppm, thereby is noted as a remarkable candidate. 1D SnO_2_ coated ZnO hybrid NWs (synthesized by solvothermal and calcination tactics) revealed sensitivity to n-Butylamine (R_a_/R_g_ = 7.4 for 10 ppm at 240 °C; response/recovery time is 40 s/80 s) with an estimated LOD of 1 ppm, thereby can be included in n-Butylamine detection [300].

Apart from NWs, nanorods were also engaged in the quantitation of volatile organic amines as detailed in the following. The sensing of Diethylamine (DEA), Trimethylamine (TMA), and TEA were demonstrated by V_2_O_5_-decorated α-Fe_2_O_3_ NRds, Au NPs decorated WO_3_ NRds, Ag NPs decorated α-MoO_3_ NRds, Cr doped α-MoO_3_ NRds, acidic α-MoO_3_ NRds, and NiCo_2_O_4_ microspheres assembled by hierarchical NRds as illustrated in Table 4 [301,302,303,304,305,306]. All these NRds were synthesized through electrospinning, calcination, wet-chemical reduction, thermal annealing, or by hydrothermal methods. Wherein, V_2_O_5_-decorated α-Fe_2_O_3_ NRds and Au NPs decorated WO_3_ NRds [293,294] demonstrated sensor utility towards DEA and TMA with the responses of 8.9 and 76.7 (response/recovery time = 2 s/40 s and 6 s/7 s, respectively) at 350 °C and 280 °C, respectively. The LODs of DEA and TMA are estimated as ~5 ppm, apart from the working temperature, it is worthy of continuing research. All other NRds [303,304,305,306] can detect the TEA at various operating temperatures (between 180 and 300 °C) as revealed in Table 4. Among them, Ag NPs decorated α-MoO_3_ NRds [295] show an exceptional sensor response to TEA (R_a_/R_g_ = 408.6 for 100 ppm at 200 °C; response/recovery time = 3 s/107 s) with a LOD of 0.035 ppm. On the other hand, NiCo_2_O_4_ microspheres assembled by hierarchical NRds [306] has a low sensor response to TEA (R_a_/R_g_ ≤ 1.5 for 50 ppm at 180 °C; response/recovery time = 49 s/54 s) with a LOD of 0.145 ppm, hence further investigations are mandatory on this material to improve the response.

**Figure 18:**
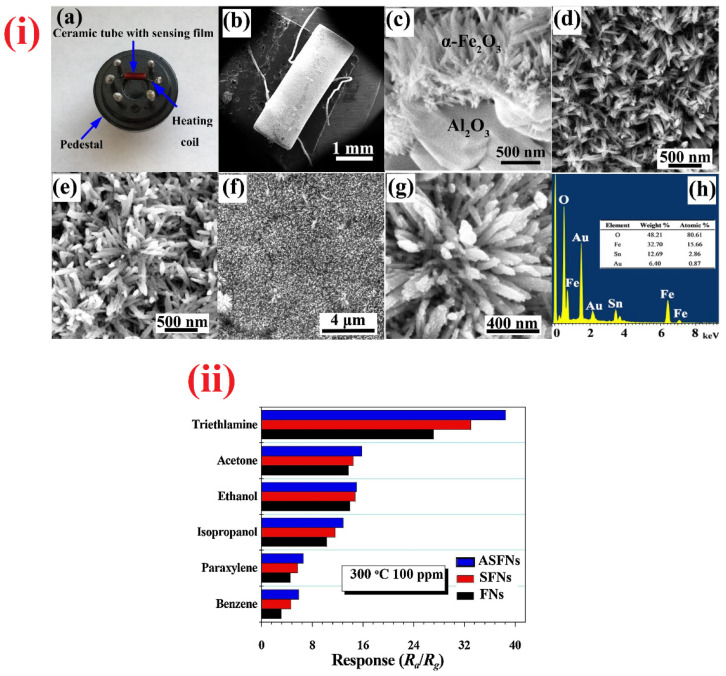
(**i**) (**a**) Gas sensor of α-Fe_2_O_3_ nanosheets fixed on an electronic bracket (**b**) Al_2_O_3_ cube covered with a film of sensing materials; (**c**) SEM image of SnO_2_ nanosheet in cross-sectional view; (**d**) SEM image of α-Fe_2_O_3_ nanoneedles (FNs) directly grown on Al_2_O_3_ tubes; (**e**) SEM images of SFNs; (**f**,**g**) SEM images of Au@SnO_2_/α-Fe_2_O_3_ nanoneedles (ASFNS) after implantation of SnO_2_ shell and Au nanoparticles; (**h**) EDS spectrum of ASFNs; (**ii**) The selectivity of different gases with same concentration at 300 °C (Reproduced with the permission from Ref. [307]).

Towards enhanced sensing of TEA, Xu and co-workers fabricated the Au@SnO_2_/α-Fe_2_O_3_ core-shell nanoneedles directly on alumina tubes via pulsed laser deposition (PLD) and DC-sputtering methods [307]. As shown in Figure 18, Au@SnO_2_/α-Fe_2_O_3_ core-shell nanoneedles displayed a higher response to TEA (R_a_/R_g_ = 39 for 100 ppm at 300 °C; response/recovery time = 4 s/203 s) than that of α-Fe_2_O_3_ nanoneedles and SnO_2_/α-Fe_2_O_3_ core-shell nanoneedles with a LOD of ~2 ppm. However, more interrogations are required to increase the response and to reduce the operating temperature.

**Figure 19:**
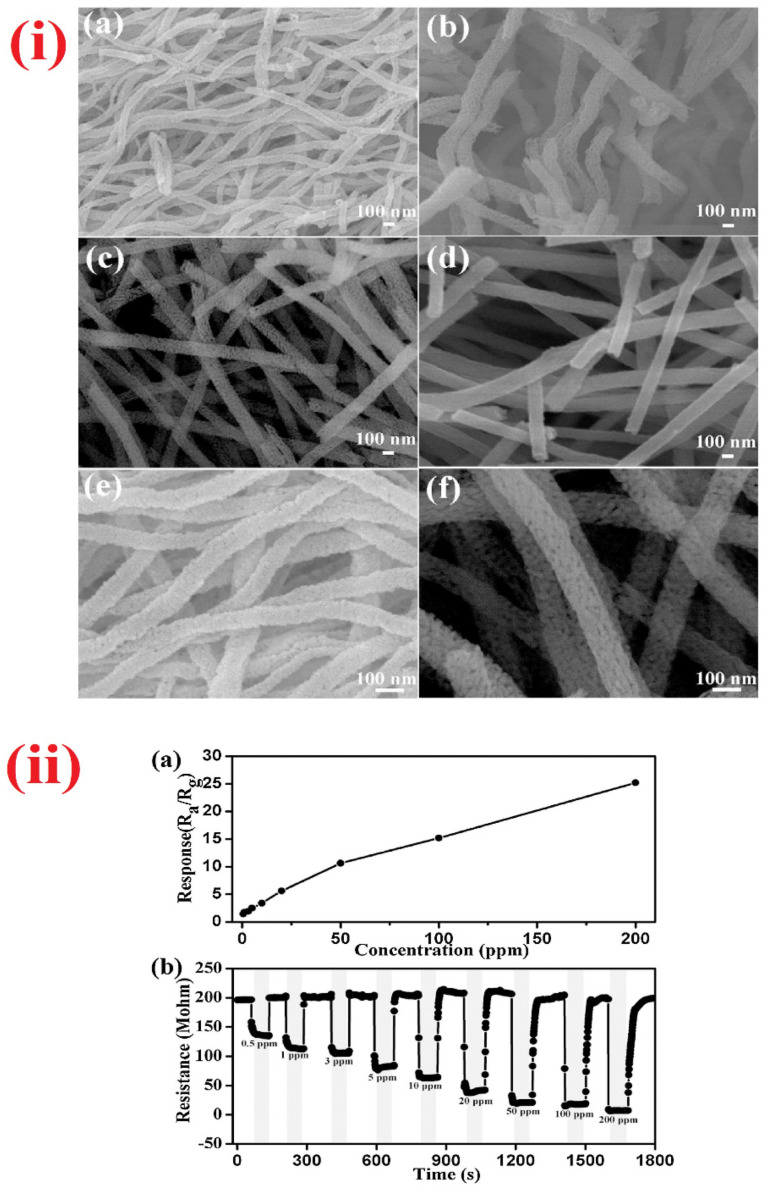
(**i**) FESEM images of S1 (**a**), S2 (**b**), S3 (**c**), S4 (**d**), high-magnification SEM images of S1 (**e**) and S3 (**f**); (Here S1–S4 represents Al_2_O_3_/α-Fe_2_O_3_ nanofibers); (**ii**) (**a**) Gas responses of the S3 sensor as a function of trimethylamine (TEA) concentrations at 250 °C. (**b**) Dynamic response-recovery curves of the sensor S3 to different concentrations of TEA at the operating temperature (Reproduced with the permission from Ref. [308]).

By means of calcination and electrospinning, nanofibers (Al_2_O_3_/α-Fe_2_O_3_ nanofibers and In_2_O_3_ hierarchical nanofibers with in situ growth of octahedron particles) were proposed by the researchers for TEA sensing studies [308,309]. As depicted in Figure 19, the Al_2_O_3_/α-Fe_2_O_3_ nanofibers [308] evidence the sensor signal even at 0.5 ppm. The sensor response to TEA is 15.19 (for 100 ppm) at 250 °C (response/recovery time = 1 s/17 s), but it is important to further reduce the working temperature in this report. In contrast, In_2_O_3_ hierarchical nanofibers with in situ growth of octahedron particles [309] revealed the highest response to TEA at 40 °C (R_a_/R_g_ = 87.8 for 50 ppm; response/recovery time = 148 s/40 min) with a LOD of ~5 ppm, thereby is noted as an excellent work. Subsequently, TiO_2_ membrane NTs and sidewall modified single-walled carbon nanotubes (SWCNTs) were employed in the detection of TMA vapor [310,311]. The flexible TiO_2_ membrane NTs showed a response of 40 for 400 ppm of TMA, but details regarding the working temperature and response/recovery time were not clear. Similarly, sidewall modified SWCNTs were demonstrated with selectivity to both ammonia and TMA at room temperature, thereby cannot be stated as a successful work. In view of this, Galstyan et al. described the DMA sensing utility of Nb doped TiO_2_ nanotubes at 300 °C as illustrated in Table 4 [312]. This work requires additional interrogations to attain a high response at low temperature.

Nanobelts such as Au NPs decorated MoO_3_ nanobelts, W doped MoO_3_ nanobelts, RuO_2_ NPs decorated MoO_3_ nanobelts, and ZnO-SnO_2_ nanobelts were developed by hydrothermal, soaking, and two step synthesis, etc., and applied in the discrimination of TMA and TEA [313,314,315,316]. Doping and decoration are the two important steps to enhance the senor signals. As shown in Table 4, these nanobelts, except the RuO_2_ NPs decorated MoO_3_ nanobelts, used in the TMA and TEA sensing interrogations require more efforts to enhance the response and to minimize the operating temperature. The RuO_2_ NPs decorated MoO_3_ nanobelts evidenced a good response (R_a_/R_g_ = 75 for 10 ppm at 260 °C; response/recovery time = 2 s/10 s) with a LOD of ~1 ppm, but the working temperature still needs further optimization. Towards TEA detection, Zhang and co-workers described the utilization of hydrothermally synthesized In_2_O_3_ nanocubes [317], which showed a high response (R_a_/R_g_ = 175 for 100 ppm at 260 °C; response/recovery time = 11 s/14 s) with a LOD of ~10 ppm, thereby is noted as an encouraging research.

As an important candidate in the TEA sensory studies, nanosheets were intensively studied by many research groups. WO_3_ NShs, Au@ZnO-SnO_2_ NShs, TiO_2_ NPs decorated CuO NShs, and Rh-SnO_2_ NShs were synthesized by precipitation, PLD, water bath treatment and solution etching methods, and surface impregnation precipitation and heat treatment method, respectively, and applied in TEA detection [318,319,320,321]. Among them, WO_3_ NShs operate at room temperature and show a sensor response of ~14 for 1000 ppm as shown in Table 4. Though this work is an impressive one, but the sensor response is not sufficiently high. On the other hand, Rh-SnO_2_ NShs displayed a great response to TEA (R_a_/R_g_ = 607.2 for 100 ppm at 325 °C; response/recovery time = 49 s/24 s) with a LOD of ~1 ppm, but further investigation is necessary to lower the working temperature. In light of this, Yan et al. reported the hydrothermally synthesized Ag modified Zn_2_SnO_4_ hexagonal nanoflakes-hollow octahedron for the enhanced sensing of TEA [322]. This material performed remarkably in terms of the sensor response (R_a_/R_g_ = 83.6 for 50 ppm at 220 °C; response/recovery time ≤ 1s/24 s) with a LOD of ~1 ppm. Subsequently, Zn_2_SnO_4_-doped SnO_2_ hollow NSPs and CeO_2_-SnO_2_ nanoflowers (by Hydrothermal synthesize) were engaged in the sensing investigations of volatile organic amines [323,324]. Wherein, Zn_2_SnO_4_-doped SnO_2_ hollow NSPs [323] is an exceptional material, which detected the phenylamine (R_a_/R_g_ = 4.53 for 50 ppm at 300 °C; response/recovery time = 10 s/4 s) with a LOD of ~1 ppm. On the other hand, CeO_2_-SnO_2_ nanoflowers [324] can be noted as an alternative in the TEA sensing at 310 °C.

**Figure 20:**
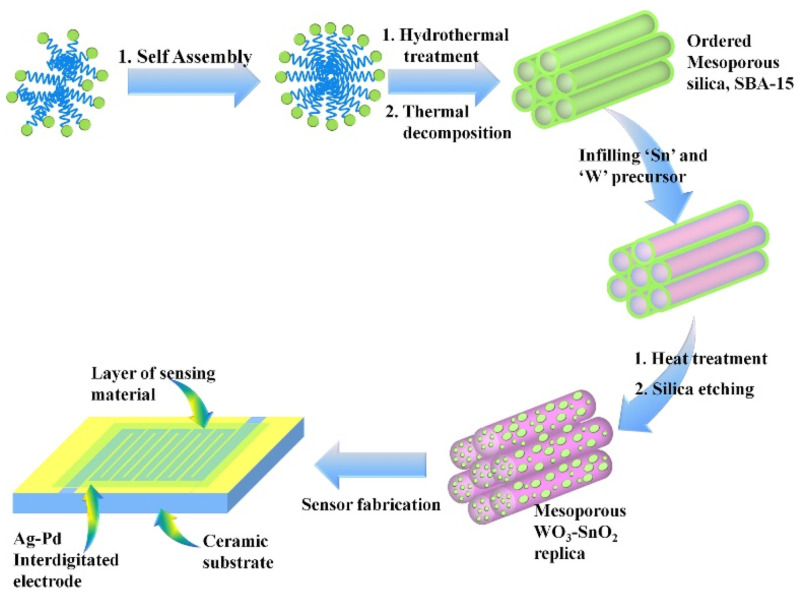
Schematic representation for nanocasting synthesis of mesoporous WO_3_–SnO_2_ by hard templating of SBA-15 (Reproduced with the permission from Ref. [325]).

Similar to diverse nanostructures, materials with porosity were vastly used in the detection of volatile organic amines as described below. WO_3_-SnO_2_ mesoporous nanostructures, CuO porous particles with diverse morphologies, In_2_O_3_ mesoporous nanocubes, CeO_2_ porous nanospheres, Au decahedrons-decorated α-Fe_2_O_3_ porous nanorods, ZnCo_2_O_4_ porous nanostructures, NiCo_2_O_4_ porous nanoplates, SnO_2_ porous thin films, Fe_2_O_3_/ZnFe_2_O_4_ porous nanocomposite, and Au-Modified ZnO porous hierarchical nanosheets were reported for the quantification of TMA or TEA as summarized in Table 4 [325,326,327,328,329,330,331,332,333,334]. Hydrothermal, solvothermal, calcination, impregnation, template methods, wet-chemical methods, etc, were used to synthesize these porous nanomaterials. Porosity scaled from nano to micro can enhance the capture of volatile amines. A schematic of WO_3_-SnO_2_ mesoporous nanostructure formation and its utilization in sensors is shown in Figure 20 [325].

Among them, In_2_O_3_ mesoporous nanocubes and Au-Modified ZnO porous hierarchical nanosheets were engaged in the sensing of TMA with decent LODs [327,334]. Similarly, CeO_2_ porous nanospheres and SnO_2_ porous thin films were reported with remarkable performance in sensing of TEA at room temperature [328,332]. In particular, SnO_2_ porous thin films [332] revealed a high sensitivity to TEA (R_a_/R_g_ = 150.5 for 10 ppm at RT; response/recovery time = 53 s/120 s) with a LOD of 0.11 ppm, thereby can be stated as excellent material towards commercialization.

**Figure 21:**
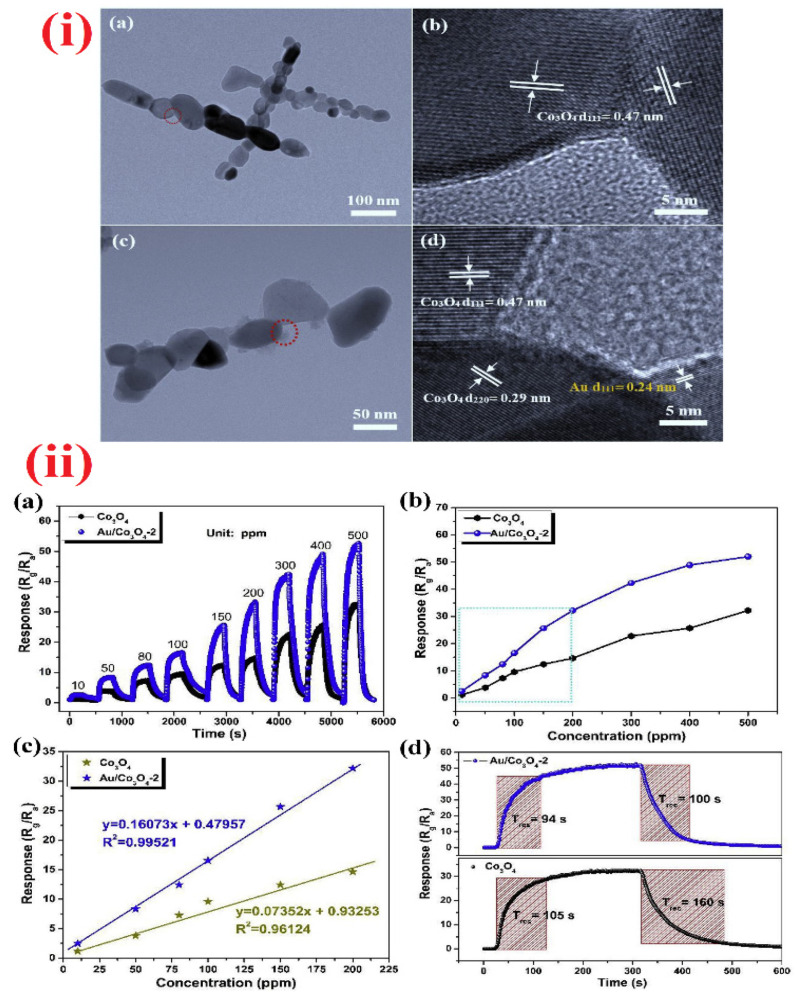
(**i**) TEM and HRTEM images of the prepared (**a**,**b**) pure and (**c**,**d**) Au/Co_3_O_4_ nanochains (Au/Co_3_O_4_-2) (**ii**) (**a**) Dynamic response-recover curves of the nanochain sensors towards various concentrations of TEA and their concentration-dependent responses within the TEA concentration range of (**b**) 10e500 ppm and (**c**)10–200 ppm; (**d**) the response and recovery curves of the sensors towards 300 ppm TEA (Reproduced with the permission from Ref. [338]).

Like other nanostructures, various hierarchical nanostructures were discussed in volatile amines detection. In light of this, α-Fe_2_O_3_ snowflake-like hierarchical nanostructure, Zn_2_SnO_4_–ZnO hierarchical nanocomposite, MoS_2_/GO 3D hierarchical nanocomposite, Au NPs decorated Co_3_O_4_ hierarchical nanochains, and WO_3_ hierarchical flower like spheres were developed and employed in TEA detection [335,336,337,338,339]. These materials were synthesized via the combination of solvothermal, annealing, hydrothermal, calcination, template route, and precipitation methods. These hierarchical nanostructures operate between 205 and 260 °C to enhance sensor responses to TEA as noted in Table 4. For example, Au NPs decorated Co_3_O_4_ hierarchical nanochains displayed a sensor response to TEA even at 10 ppm as shown in Figure 21 [338]. However, among the aforementioned hierarchical nanomaterials, Zn_2_SnO_4_–ZnO hierarchical nanocomposite [336] revealed a high response to TEA (R_a_/R_g_ = 175.5 for 100 ppm at 200 °C; response/recovery time = 12 s/25 s) with a LOD of 0.4 ppm, thereby can be noted as an inspiring research in TEA sensors.

Apart from the utilization of distinct nano-architectures, numerous diversified nano-shaped materials were employed in the quantitation of volatile organic amines. In view of this, ZnO/Au hemishperical nanostructure, SnO_2_/Au/Fe_2_O_3_ nanoboxes, Au decorated ZnO nest-like nanostructure, Pd doped ZnO agaric like nanostructure, Co_3_O_4_@MnO_2_ shish-kebab like nanostructure, and Au@ZnO core-shell nanostructure were demonstrated in TEA and aniline detection as noted in Table 4 [340,341,342,343,344,345]. In particular, Pd-doped ZnO agaric like nanostructure [343] was used in aniline sensing with a high response (R_a_/R_g_ = 182 for 100 ppm at 280 °C; response/recovery time = 29 s/23 s) with a LOD of 0.5 ppm, thereby is noted as a distinct research. Among the other materials in TEA discrimination, Au@ZnO core-shell nanostructure [345] is an exceptional material with excellent sensor performance at 50 °C (R_a_/R_g_ = 12.2% for 5 ppm; response/recovery time is 27 s/46 s) and a LOD of ~1 ppm. In parallel to diverse nanostructures, composite materials without any structural modifications were also utilized in TEA and TMA sensors. Materials such as ZnO/ZnFe_2_O_4_ composites, Au/Co_3_O_4_/W_18_O_49_ hollow composite nanospheres, α-Fe_2_O_3_@α-MoO_3_ composite, CuO/ZnO 3D diamond shaped MOF, rGO decorated W-doped BiVO_4_ hierarchical nanocomposite, and Au@MoS_2_ nanocomposite were effectively consumed in TEA or TMA detection [346,347,348,349,350,351]. Herein, ZnO/ZnFe_2_O_4_ composite displayed a good sensitivity to TEA under irradiation (R_a_/R_g_ ≥ 9 for 500 ppm at 80 °C) and requires additional interrogations to enhance the response [346]. Similarly, p-n CuO/ZnO 3D diamond shaped MOF composite authenticated the selectivity to TEA and methanol at 220 °C and 260 °C, respectively [349]. However, this composite showed a better response to TEA (R_a_/R_g_ ≥ 400 for 500 ppm; response/recovery time = 11 s/~60 min) than that of methanol with a LOD of 0.175 ppm. However, this research still needs further work. Compared to the other composite materials, Au@MoS_2_ nanocomposite is a unique material with its sensory response to TEA at 30 °C (R_a_/R_g_ = 44 for 50 ppm; response/recovery time = 9 s/91 s) and a LOD of ~2 ppm as noted in Table 4 [351].

## 7. Volatile Hydrocarbons Detection by Distinct Nanostructures

Following the similar mechanism mentioned earlier in acetone and alcohols discrimination, hydrocarbons sensors were reported using various nanostructured materials. Nanoparticles were oftenly investigated by many researchers. Chen et al. reported Ag-LaFeO_3_ NPs developed via two different tactics like MIP and lotus-leaf templated synthesis and engaged in xylene detection at 99 °C and 125 °C, correspondingly [352,353]. As noted in Table 5, Ag-LaFeO_3_ NPs synthesized via MIP tactic seems to be good in terms of sensor response (R_a_/R_g_ = 36.2 for 5 ppm at 99 °C; response/recovery time = 114 s/55 s) with a LOD of <1 ppm, thereby Ag-LaFeO_3_ NPs can be attested as an inspiring candidate in xylene sensors. Similar to above studies, Au loaded ZnO NPs and cobalt porphyrin (CoPP)-functionalized TiO_2_ NPs were described for the quantitation of xylene and BTX (Benzene, Toluene and Xylene) vapors at 377 °C and 240 °C, respectively [354,355]. Wherein, cobalt porphyrin (CoPP)-functionalized TiO_2_ NPs showed a high response (R_a_/R_g_ ≥ 5 for 10 ppm at 240 °C; response/recovery time = 40 s/80 s) with a LOD of 0.005 ppm [355]. However, further studies are needed to minimize the operating temperatures in both cases. Other than NPs, Park and co-workers described the utility of In-doped ZnO Quantum dots (QDs) towards the sensing of acetylene gas at 400 °C with the LOD down to sub-ppm level (0.1 ppm) [356]. However, the working temperature in this report was high, which requires more work. In view of this, Xu et al. exploited the BTEX (Benzene, Toluene, Ethyl benzene, and Xylene) detection by MOF derived nanocrystals at room temperature [357]. However, anti-interference studies with this material is still in question, thereby cannot be attested as excellent work.

**Figure 22:**
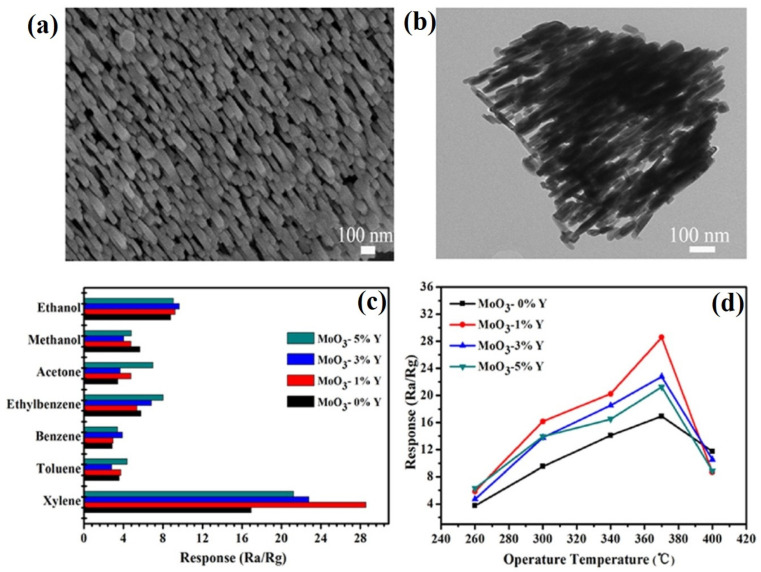
(**a**,**b**) The SEM and TEM images of nanoarrays; (**c**) Response curve of samples to 100 ppm different Gases at the working temperature of 370 °C; (**d**) Response curve of samples to 100 ppm xylene at the different working temperature (Reproduced with the permission from Ref. [363]).

To discriminate the volatile toluene, α-Fe_2_O_3_/SnO_2_ NW arrays and Pt NPs sensitized Si NW-TeO_2_ NWs (opted from ultrasonic spray pyrolysis, hydrothermal, and sputtering tactics) were proposed with sensor responses >40 at 90 °C and 200 °C, respectively, as noted in Table 4 [358,359]. Such NWs-based sensory research is noted as an innovative one. Pd NPs decorated TiO_2_ NRds were employed in the sensing of liquefied petroleum gas (LPG), but further interrogations are required for authentication [360]. Following above work, Lee et al. fabricated the cobalt porphyrin (CoPP)-ZNO NRds towards the detection of Toluene (R_a_/R_g_ = 3.3 for 10 ppm) and estimated a LOD of 0.002 ppm [361]. Moreover, this material can operates in the 0 to 85% humid conditions, thereby attest as a nice work. Qin and co-workers described the Y-doped or undoped α-MoO_3_ nanoarrays towards the enhanced sensing of Xylene at 370 °C as noted in Table 5 [362,363]. As shown in Figure 22, authors conformed the 1% Y doping over α-MoO_3_ nanoarrays displayed an improved sensor response than that of other doping concentrations or non-doping (0, 3%, and 5%). However, both works do not merit the commercialization due to the higher operating temperature.

Nanofibers, such as MOF-driven metal-embedded metal oxide (Pd@ZnO-WO_3_) nanofibers, V_2_O_5_ nanofibers, and Pd functionalized SnO_2_ nanofibers were engaged in the determination of toluene, xylene, and butane, correspondingly [364,365,366]. Pd@ZnO-WO_3_ nanofibers showed a high response to toluene (R_a_/R_g_ = 22.22 for 1 ppm at 350 °C; response/recovery time ≤ 20 s/not available) with a LOD of 0.1 ppm, thereby become an impressive candidate apart from the working temperature [356]. Subsequently, V_2_O_5_ nanofibers were noted as highly remarkable material to be employed in xylene detection at room temperature (R_a_/R_g_ = 191 for 500 ppm at RT; response/recovery time = 80 s/50 s (for 100 ppm)) with an estimated LOD of ~ 5 ppm [357]. Similar to above research, Pd functionalized SnO_2_ nanofibers [366] used in the discrimination of butane at 260 °C (see Table 5), but its sensor response needs to be improved with further investigations. As an inclusion to the VOC sensory research, nanotubes (Pt-decorated CNTs, 3D TiO_2_/G-CNTs and hierarchical NiCo_2_O_4_ NTs) were demonstrated towards toluene or xylene discrimination [367,368,369]. As seen in Table 5, TiO_2_ NPs decorated G-CNTs seems to be an excellent candidate towards toluene detection at room temperature with a calculated LOD of 0.4 ppm [368].

**Figure 23:**
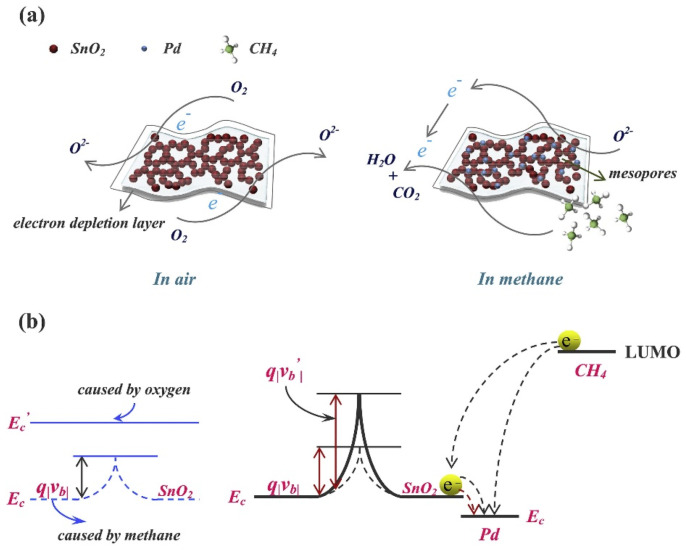
(**a**,**b**) Schematic diagram of the sensing reaction mechanism of the Pd-SnO_2_ composite nanoporous structure (Reproduced with the permission from Ref. [380]).

**Figure 24:**
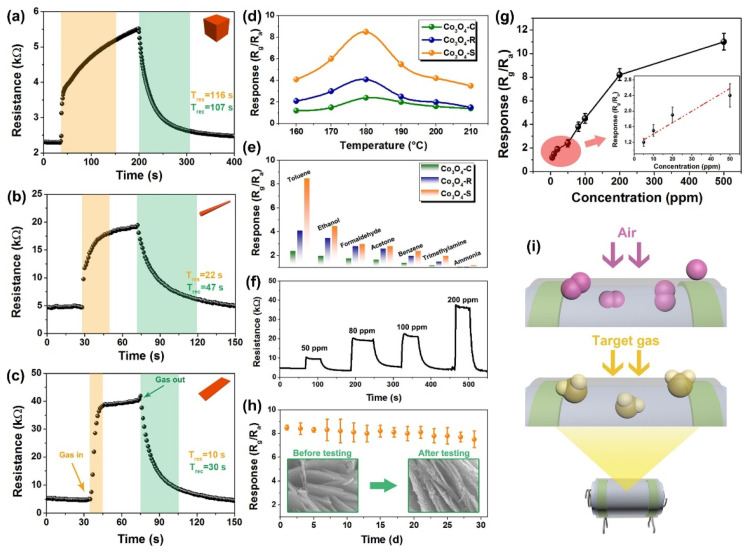
Resistance curves of the sensors based on (**a**) Co_3_O_4_-C, (**b**) Co_3_O_4_-R, (**c**) Co_3_O_4_-S towards 200 ppm toluene at 180 °C, respectively; (**d**) responses of three sensors towards 200 ppm toluene at different working temperatures; (**e**) responses of three sensors to 200 ppm different target gases at 180 °C; (**f**) dynamic resistance curve of the sensor based on Co_3_O_4_-S to different concentration of toluene; (**g**) relationship between response and toluene concentration; (**h**) response and morphology stability of Co_3_O_4_-S-based sensor to 200 ppm toluene during 30 days (measurement number = 3); (**i**) schematic of sensor exposed to air and target gas (Reproduced with the permission from Ref. [385]).

Nanobelts (Fe doped MoO_3_ nanobelts and Au decorated ZnO/In_2_O_3_ belt-tooth nanostructure), nanocages (ZnO/ZnCo_2_O_4_ hollow nanocages), nanosheets (Au functionalizedWO_3_·H_2_O NShs, porous h-BN 3D NShs, and Nb-doped NiO NShs), nanoflakes (CdO hexagonal nanoflakes and ZnO-CeO_2_ triangular nanoflakes), and nanospheres (ZnFe_2_O_4_ NSPs and Pt doped CoCr_2_O_4_ hollow NSPs) were demonstrated to volatile hydrocarbons quantitation by the researchers as noted in Table 5 [370,371,372,373,374,375,376,377,378,379]. Among them, Nb-doped NiO NShs and CoCr_2_O_4_ hollow NSPs [375,379] are highly notable with their sensor responses to xylene vapor (R_a_/R_g_ = 335.1 for 100 ppm at 370 °C and R_a_/R_g_ = 559 for 5 ppm at 275 °C, respectively) with LODs down to sub-ppm level (0.002 ppm and 0.0187 ppm, correspondingly). In light of this, involvement of porous nanostructures is anticipated due to the high adsorption nature through the pores [374]. Yao et al. discussed the pore-effect of Pd-SnO_2_ nanoporous composite [380] for the capture and sensing of methane gas as illustrated in Figure 23. The material shows reasonable sensor response (see Table 5) in methane sensory research. In view of this, p-n Co_3_O_4_–TiO_2_ mesoporous hierarchical nanostructures were elaborated in the sensing of xylene at an operating temperature of 115 °C [381]. In which, the response is well enough (R_a_/R_g_ = 113 for 50 ppm; response/recovery time = 130 s/150 s) due to the pore-effect of the material. In parallel to utilization of distinct nanostructures in volatile hydrocarbons discriminations, many hierarchical nanomaterials were also employed as described below. Hierarchical nanostructures like Au loaded MoO_3_ hollow NSPs, Pt-SnO_2_ hollow NSPs, NiO/NiMoO_4_ NSPs, Co_3_O_4_, and WO_3_ were demonstrated in the sensing of toluene, xylene, methane, or acetylene at various temperature (180–340 °C) as noted in Table 5 [382,383,384,385,386]. Among them, hierarchical nanostructure of Co_3_O_4_ displayed a sensor response to toluene at 180 °C even after one month, thereby is noted as a reliable system [385]. In this work, authors described the sensory performance of cube shaped Co_3_O_4_ (C-Co_3_O_4_), rod shaped Co_3_O_4_ (R- Co_3_O_4_), and sheet shaped (S- Co_3_O_4_) nanostructures synthesized via hydrothermal tactic. Wherein, sheet shaped (S- Co_3_O_4_) hierarchical nanostructure showed a higher response than that of others as shown in Figure 24.

In addition, PbS NPs decorated CdO necklace like nanobeads were discussed in the quantitation of LPG gas at RT by Sonawane and co-workers [387]. Nevertheless, further investigation is necessary to authenticate the material’s reliability. In light of this, Au loaded TiO_2_ hedgehog-like nanostructure was proposed to detect xylene at 375 °C, thereby can be included as an additional candidate [388]. These materials were synthesized by hydrothermal method followed by simple isometric impregnation route. They are required to be optimized to minimize the working temperature during the sensor studies.

Similar to microstructure-based volatile hydrocarbon vapors quantification (example: Sn^2+^ doped NiO microspheres in Xylene detection with sub-ppm LOD) [389], nanocomposites, such as Pd/PdO/S-SnO_2_ nanocomposite film, rGO/Co_3_O_4_ nanocomposite, WO_3_ decorated TiO_2_ NPs nanocomposite, BGQD/Ag–LaFeO_3_ nanocomposite, Ag/Bi_2_O_3_ nanocomposite, AgO loaded LaFeO_3_ nanocomposite, CuO NPs-Ti_3_C_2_Tx MXene nanocomposite, and Graphene/SnO_2_ NPs nanocomposite were effectively applied in the detection of hydrocarbons as detailed in Table 5 [390,391,392,393,394,395,396,397]. Wherein, Ag/Bi_2_O_3_ nanocomposite and Graphene/SnO_2_ NPs nanocomposite were found to perform remarkably in terms of their operation temperature (at room temperature) [394,397]. Recently, Luo et al. described the sensitivity of a polymer-SWCNTs composite toward BTX with an exceptional LOD of 5 ppm [398]. However, this work also depends on the polymer properties and requires more detailed investigations.

## 8. Nanostructures in other VOCs Determinations

**Figure 25:**
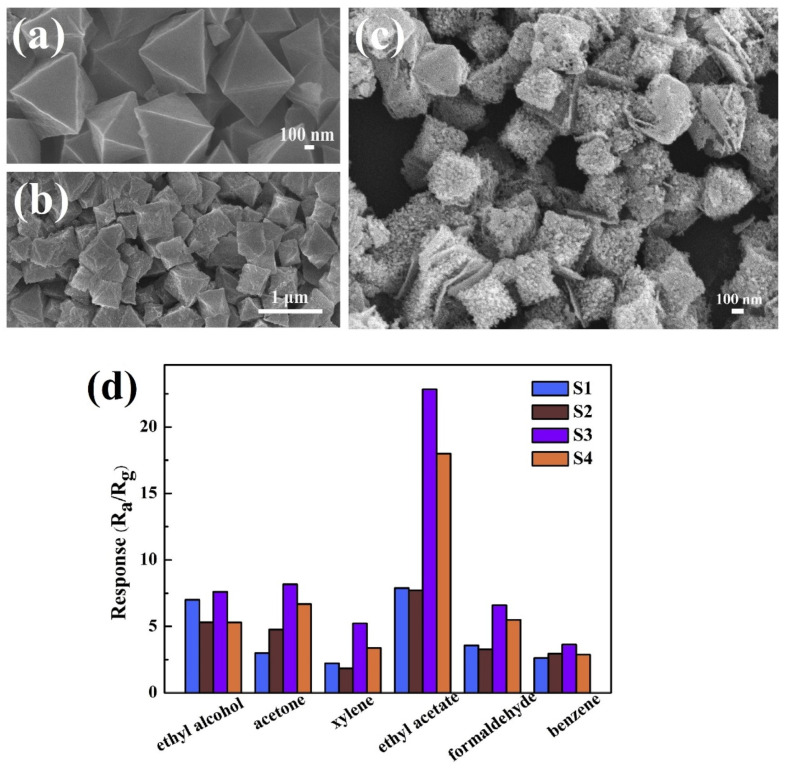
(**a**) Typical FESEM images of MIL-53 nano-octahedrons; (**b**) SEM images of MIL-53/Pr–Fe hydroxide precursor; (**c**) typical FESEM image of pure core-shell α-Fe_2_O_3_ nano-octahedrons; (**d**) Selectivity of four sensors upon exposure to 100 ppm various interfering gases at 255 °C (S1) and 206 °C (S2–S4), respectively, (reproduced with the permission from Ref. [402]).

Apart from the quantitation of acetone, alcohols, aldehydes, amines, and hydrocarbons, nanomaterials were also consumed in the discrimination of other VOCs as detailed in this section. For instance, Ma et al. reported the acetic acid sensing properties of Co-doped LaFeO_3_ nanofibers [399]. The fabricated nanofibers detected the acetic acid at 130 °C than that of other interferences (ethanol, methanol, acetone, N,N′-dimethylformamide (DMF), ammonia, and benzene). This work requires further investigations to identify various sensory details. Mesoporous tungsten oxides with crystalline framework were utilized in the highly selective detection of foodborne pathogen “3-hydroxy-2-butanone” and proposed in food safety by Zhu and co-workers [400]. Compared to all other competing species at high concentration (50 ppm), the material displayed a high response to 3-hydroxy-2-butanone at low concentration (R_a_/R_g_ ≥ 25 for 5 ppm) at 290 °C with response/recovery time of <1 min and a LOD of 0.1 ppm. The porosity of this material plays a vital role in the sensory studies with similar mechanism proposed in acetone. This is an excellent and representative work in food safety. Aqueous electrochemical-based detection of chloroform was proposed by Hamid and co-workers [401]. Wherein, Fe_2_O_3_ NPs decorated ZnO NRds were used in this work, which showed linear response from 10 µM to 10 mM with a calculated LOD of 0.6 µM. However, this material needs to be investigated to optimize the resistance-based sensor responses. For example, MOF derived core-shell PrFeO_3_ functionalized α-Fe_2_O_3_ nano-octahedrons were employed in the quantitation of ethyl acetate at 206 °C as seen in Figure 25 [402]. For 100 ppm ethyl acetate, the sensor response reached 22.85 with response/recovery time of <15 s with LOD down to 1 ppm. Therefore, this work is noted as an excellent one towards ethyl acetate discrimination in the presence of competing species (ethanol, acetone, xylene, formaldehyde, and benzene).

Gao and his research team discussed the detection ability of ZnO NRds bundles towards three kinds of abused drugs (C_10_H_15_NO, C_9_H_13_NO and C_8_H_9_NO_2_; 100 ppm) at 252 °C via the similar mechanism in VOCs detection [403]. These ZnO NRds bundles were synthesized by hydrothermal method, and upon exposure to those abused drugs vapors, the sensor responses were higher (>40) than that of other interferences. Firstly, the n-type ZnO NRds bundles adsorb O_2_ molecules in air to capture free electrons in conduction band (at the device surface) and convert them to adsorbed oxygen species (O^2−^, O_2_^−^, O^−^). Secondly, decrease in the number of electrons in the conduction band leads to the formation of a depletion layer, which increases resistance in ZnO. Consequently, the electrons are released back to conduction band upon interaction with target drug vapors, which affects the depletion layer and the resistance of ZnO. The changes in resistance can provide sensor responses of interactions with target drug vapor. Moreover, this work performed quite well at high humid condition (>90% RH). In a similar fashion, Ag–ZnO–SWCNT field effect transistor (FET) device demonstrated its sensing performance to organophosphorus pesticide “methyl parathion” with linear response from 1 × 10^−16^ to 1 × 10^−4^ M and a LOD of 0.27 × 10^−16^ M [404]. The response and recovery time of this sensor is 1 s and 3 s, respectively. Moreover, this work also attested by real time analysis in rice and soil, thereby is noted as good innovation.

A FET device based on electrospun InYbO nanofibers was proposed towards the sensing of DMF at room temperature by Chen and co-workers [405]. The sensor showed remarkable response (R_a_/R_g_ = 89) to 2 ppm DMF, with an estimated LOD of 0.00118 ppm. Moreover, it delivered fast response/recovery time of 36 s/67 s, thereby is noted as encouraging research with a wide scope. In view of this, MgGa_2_O_4_/graphene composites were synthesized by simple hydrothermal route and utilized in the sensing of acetic acid at room temperature [406]. At 0.1 wt% graphene, the material displayed a high sensor response (R_a_/R_g_ = 363.3 for 100 ppm; response/recovery time = 50 s/35 s) with a calculated LOD of 0.001 ppm. Therefore, it is noted as an exceptional material and can be consumed in acetic acid sensory studies in future.

## 9. Advantages and Limitations

Consumption of distinct nanostructured materials in VOCs detection also has a few advantages and limitations as noted below.

1. By tuning the nanostructural features, materials with similar compositions are able to detect diverse VOC analytes.
2. Modulation of nanostructures might be able to tune the availability of surface area to enhance adsorption of VOC analytes, thereby the sensor response can be improved significantly.
3. By adopting different nanostructures, the operating temperature for the sensing of specified VOC can be reduced or lowered to room temperature.
4. Divergent nanostructures formation via the combination of diverse materials and their decoration or doping or functionalization may enhance/tune the conducting and charge/electron transport properties, which can help the device to detect specific VOC from other competing species.
5. Utilization of known and easily fabricated semiconducting materials with diverse nanostructures may led to the generation of cost-effective and reliable devices with social impact.
6. The less toxic nature of some semiconducting materials is noted as an advantage and can be implemented in upcoming health care devices.
7. Synthesis of majority of diverse nanostructures seems to be more complicated with involvement of multiple synthetic tactics, such as hydrothermal, CVD, impregnation, electrospinning, etc. The requirement of processing at high temperature further increases the production cost.
8. Reliability of numerous diverse semiconducting nanostructures to specific VOC is still in question due to the higher operating temperature.
9. Reports on temperature dependent multiple analyte sensors by a few nanostructured materials are still not convincing with the interference studies; therefore, applicability of those materials is limited and requires more interrogations.
10. Fabrication of diverse nanostructured materials is also limited by the sophisticated clean room atmosphere and characterizations using costly equipment, such as SEM, TEM, electrochemical instruments, etc.
11. Majority of reported nanostructures are still not authenticated by real time applications, which limits the operation of those devices in VOC detection.
12. In general, porous nanostructures display the high/low responses to VOCs due to their pore-effect but are limited in operation by their uneven results.
13. Stability of sensory reports is considerably affected by high humid conditions and is restricted in real time applications.

## 10. Conclusions and Perspectives

This review gives a concise summary of the diverse nanostructured material-based detection of VOCs (more than 340 references that have been published since 2016), such as acetone, alcohols, aldehydes, amines, hydrocarbons, and other volatile organic compounds. The underlying mechanism of those temperature tuned semiconducting device-based assays of VOCs is also discussed in brief with given advantages and limitations. Moreover, the VOC sensory utilities of diverse nanostructures and nanocomposites with p-p, p-n, n-n heterojunction structures are also presented. Wherein, numerous inorganic-nanostructured materials such as metal oxides, perovskites and composited structures are currently noted as effective candidates towards VOCs detection. Further, majority of reports mainly follows the similar trend to detect the aforementioned VOCs. In addition to those sensory details, following points also require much attention.

1. Synthetic complications on the development of semiconducting materials towards VOCs sensors must be reduced with respect to cost-effect and reliability.
2. Justification regarding the role of semiconducting property in the sensing studies seems to be deficient in some reports, which requires more investigations.
3. Majority of the reports did not provide any theoretical or in-depth explanation regarding, “Why the attested nanostructure becomes more specific to the certain target?” Therefore, extensive research must be done in future.
4. Utilization of diverse nanostructures with similar material compositions towards different VOCs still needs more interpretations.
5. Numerous VOCs sensors operate at higher temperature, thereby optimization research should be conducted to make the devices operable at low temperature or even room temperature.
6. Studies on how to resolve the factors that affecting the sensor response (such as humidity, interferences, temperature, etc.) are necessary.
7. So far, majority of VOC sensors make use of metal-oxide nanostructures, thereby implementation of devices with emerging halide perovskite nanomaterials can be much anticipated in VOCs detection.
8. Many reports describe the dopant and composited materials tuned sensory responses towards specific VOCs, but there is no-valid information regarding the efficacy and the role of such dopants or composited materials on the VOCs sensing, which requires clarification.
9. Enormous amount of reports are available for acetone and alcohols chemiresistor sensors, thus upcoming researchers must focus towards commercialization rather than simply developing the new materials.
10. Due to the high toxic effect of aldehydes and amines over the eco-systems, chemiresistor sensors coupled with eco-friendly instrumental set up is requires much focus.
11. Mainstream of BTEX assays by distinct nanostructures are still need to be tuned towards specific target due to their ineffectiveness towards mixed analytes.
12. Detection of other toxic VOCs (such as carbon tetrachloride (CCl_4_), chloroform (CHCl_3_), phosgene, etc.) needs much attention in future.
13. Standardized procedure is become mandatory to attain the specific nanostructures and its VOC sensing performance.
14. Development of stable and commercial devices in the determination of VOCs is still in demand, therefore, much attention is required for commercialization.
15. More research is necessary to justify the exact production cost of reliable devices in VOCs detection with social importance.

Though mechanistic aspects of VOCs detection have been clarified by numerous reports; however, theoretical and in-depth discussions regarding charge/electron transport in semiconducting properties are yet to be improved. As an essential research in health care innovations, many scientists are currently trying to develop and commercialize cost-effective devices towards specific VOC targets, which may improve the safety of the healthcare and food products in future.

## Figures and Tables

**Table 1:** Detection concentration, response/recovery time, operating temperature (Temp.) and limits of dection (LODs) to acetone gas by diverse nanostructured material.

Materials/Nanostructure	Analyte/Concentration	Gas Response (S = R_air_/R_gas_)	Response/Recovery	Temp.	LOD	Ref
TiO_2_/nanoparticles	Acetone/500 ppm	9.19	10 s/9 s	270 °C	0.5 ppm	[59]
α-Fe_2_O_3_/nanoparticles	Acetone/100 ppm	8.8	NA	340 °C	5 ppm	[60]
Mn doped ZnO/nanoparticles	Acetone/2 ppm	3.7	17 s/NA	340 °C	1.8 ppm	[61]
Pt-decorated Al-doped ZnO/nanoparticles	Acetone/10 ppm	421	2.9 s/440 s	450 °C	~0.1 ppm	[62]
Al doped ZnO/nanoparticles	Acetone/10 ppm	11.8	11 s/793 s	500 °C	0.01 ppm	[63]
B-TiO_2_@Ag/nanoparticles	Acetone/50 ppm	68.19	12 s/41 s	250 °C	0.887 ppm	[64]
La_1-x_Y_x_MnO_3-⸹_/nanoparticles	Acetone/500 ppm	27.2	NA	300 °C	NA	[65]
Bi_1-x_La_x_FeO_3_/nanoparticles	Acetone/0.05 ppm	8	15 s/13 s	260 °C	0.05 ppm	[66]
SmFe_1−x_Mg_x_O_3_/nanocrystals	Acetone/0.5 ppm	7.16	32 s/8 s	220 °C	0.01 ppm	[67]
WO_3/_nanocrystals	Acetone/0.25 ppm	3.8	4 s/5 s	320 °C	0.0075 ppm	[68]
TiO_2_-5Rh/nanocrystals	Acetone/50 ppm	9.6	NA	300 °C	10 ppm	[69]
Co_3_O_4_ NPs attached SnO_2_/nanowires	Acetone/50 ppm	70	NA/122 s	300 °C	0.5 ppm	[70]
self-assembled monolayer (SAM) functionalized ZnO/nanowires	Acetone/50 ppm	170 & 90	2 min/24 min & 3 min/29 min	300 °C	0.5 ppm	[71]
Branched p-Cu_x_O @ n-ZnO/nanowires	Acetone/5–50 ppm	3.39–6.38	62 s/90 s	250 °C	~5 ppm	[72]
Cr doped ZnO single-crystal/nanorods	Acetone/70 ppm	70	NA	300 °C	~10 ppm	[73]
SnS_2_/nanorods	Acetone/ 10 ppm	25	NA	300 °C	Down to sub-ppm	[74]
Au@ZnO & Pd@ZnO/nanorods	Acetone/100 ppm	44.5 & 31.8	8 s/5 s & 17 s/11 s	150 °C	0.005 ppm	[75]
α-Fe_2_O_3_-NiO/nanorods	Acetone/100 ppm	290	28 s/40 s	280 °C	~5 ppm	[76]
Ag-doped ZnO/nanoneedles	Acetone/200 ppm	30.233	10 s/21 s	370 °C	~10 ppm	[77]
La-doped SnO_2_/nanoarrays	Acetone/200 ppm	69	6–12 s/20 s	290 °C	5 ppm	[78]
α-Fe_2_O_3_-SnO_2_/nanoarrays	Acetone/1 ppm	5.37	14 s/70 s	340 °C	1 ppm	[79]
ZnTiO_3_/nanoarrays	Acetone/12.5 ppm	78 & 94	117 and 141 s/99 and 131 s & 75 and 81 s/50 and 69 s (dark & light)	45 °C & 350 °C	0.01 & 0.09 ppm	[80]
Ag-decorated SnO_2_/nanofibers	Acetone/50 ppm	40	6 s/10 s	160 °C	5 ppm	[81]
PrFeO_3/_nanofibers	Acetone/200 ppm	141.3	7 s/6 s	180 °C	10 ppm	[82]
Pt-ZnO-In_2_O_3_/nanofibers	Acetone/100 ppm	57.1	1 s/44 s	300 °C	0.5 ppm	[83]
Au@WO_3_-SnO_2/_nanofibers	Acetone/10 ppm	196.1	~2 min (for both)	150 °C	<0.5 ppm	[84]
Au functionalized In-doped ZnSnO_3_/nanofibers	Acetone/50 ppm	19.3	10 s/13 s	200 °C	~10 ppm	[85]
ZnO/nanofibers	Acetone/100 ppm	50–124	65–130 s/75–135 s	250 °C	NA	[86]
Ru-doped SnO_2/_nanofibers	Acetone/100 ppm	118.8	1 s/86 s	200 °C	~0.5 ppm	[87]
Pd-SnO_2_/nanotubes	Acetone/5 ppm	93.55	NA	350 °C	<1 ppm	[88]
PdO@ZnO-SnO_2_/nanotubes	Acetone/1 ppm	5.06	20 s/64 s	400 °C	0.01 ppm	[89]
α-Fe_2_O_3_ nanorods-MWCNTs/nanotubes	Acetone/100 ppm	38.7	2 s/45 s	225 °C	0.5 ppm	[90]
Pt-decorated CuFe_2_O_4_/nanotubes	Acetone/100 ppm	16.5	16 s/299 s	300 °C	~5 ppm	[92]
WO_3_–SnO_2_/nanotubes	Acetone/50 ppm	63.8	NA	275 °C	0.05 ppm	[93]
ZnO-Decorated In/Ga Oxide/nanotubes	Acetone/100 ppm	12.7 & 27.1	6.8 s/6.1 s & 11.8 s/11.6 s	300 °C	0.2 ppm	[94]
Y doped SnO_2_/nanobelts	Acetone/100 ppm	11.4	9–25 s/10–30 s	210 °C	0.9024 ppm	[95]
Eu doped SnO_2_/nanobelts	Acetone/100 ppm	8.56	15 s/19 s	210 °C	0.131 ppm	[96]
Co_3_ O_4/_nanocubes	Acetone/500 ppm	4.88	2 s/5 s	240 °C	~10 ppm	[97]
Ag-ZnSnO_3_/nanocubes	Acetone/100 ppm	30	2 s/3 s	280 °C	1 ppm	[99]
ZnO−CuO/nanocubes	Acetone/1 ppm	11.14	NA	200 °C	0.009 ppm	[100]
NiFe_2_O_4_/nanocubes	Acetone/1 ppm	1.9	8 s/40 s	160 °C	0.52 ppm	[101]
NiO/ZnO/nanocubes	Acetone/200 ppm	58	24 s/133 s	340 °C	~10 ppm	[102]
MOF derived-ZnO/ZnFe_2_O_4_/nanocubes	Acetone/5 ppm	9.4	5.6 min/6 min	250 °C	<0.5 ppm	[103]
PdO@Co_3_O_4_/nanocages	Acetone/5 ppm	2.51	NA	350 °C	0.1 ppm	[104]
ZnO/ZnFe_2_O_4_/nanocages	Acetone/100 ppm	25.8	8 s/32 s	290 °C	1 ppm	[105]
PdO-NiO/NiCo_2_O_4/_nanocages	Acetone/100 ppm	6.7	<20 s/<30 s	210 °C	NA	[106]
Ag@CuO- TiO_2_/nanocages	Acetone/100 ppm	6.2	56 s/9 s	200 °C	~1 ppm	[107]
Co_3_O_4_/ nanosheets	Acetone/100 ppm	16.5	NA	111 °C	~5 ppm	[108]
ZnO_/_nanosheets	Acetone/5 ppm	6.7	<60 s (for both)	300 °C	<5 ppm	[109]
SnO_2_/Fe_2_O_3_/nanosheets	Acetone/10 ppm	9.8	0.8 s/3.4 s	260 °C	NA	[110]
NiO/nanosheets	Acetone/150 ppm	>90	80 s/82 s	200 °C	0.83 ppm	[111]
Fluorine doped TiO_2_/nanosheets	Acetone/800 ppm	17.42	162 s/220.5 s	25 °C	~25 ppm	[112]
Nb-doped ZnO & ZnO/nanowalls	Acetone/100 ppm	84.62 & 89.13	NA	200 °C & 200 °C	<20 ppm	[113]
CuO/nanowalls	Acetone/500 ppm	4	82 s (50 ppm)/NA	320 °C	2 ppm	[114]
NiO/nanowalls	Acetone/10 ppm	>30	NA	250 °C	0.2 ppm	[115]
α-MoO_3_/nanoflakes	Acetone/10–100 ppm	NA	NA	150 °C	NA	[117]
SnS/nanoflakes	Acetone/100 ppm	>1000	3 s/14 s	100 °C	<5 ppm	[118]
NiO/ZnO/nanospheres	Acetone/100 ppm	29.8	1 s/20 s	275 °C	Down to sub-ppm	[122]
WO_3_-SnO_2_/nanospheres	Acetone/50 ppm	~8 & 16	16 s/12 s & 15 s/11 s	240 °C	~50 ppm	[123]
Na:ZnO/nanoflowers	Acetone/100 ppm	3.35	18.2 s/63 s	NA	0.2 ppm	[124]
ZnO_/_nanoflowers	Acetone/100 ppm	2900 & 300	5 s/NA	365 °C & 248 °C	<20 ppm	[126]
RuO_2_-modified ZnO/nanoflowers	Acetone/100 ppm	125.9	1 s/52 s	172 °C	<25 ppm	[127]
Au NPs-Fe_2_O_3_/porous nanoparticles	Acetone/10 ppm	6.1	5 s/20 s	200 °C	0.132 ppm	[128]
Au/ZnO/porous nanoparticles	Acetone/1 ppm	17.1	231 s/215 s	275 °C	<0.1 ppm	[129]
ZnFe_2_O_4_/porous nanorods	Acetone/100 ppm	52.8	1 s/11 s	260 °C	<10 ppm	[130]
α-Fe_2_O_3_/SnO_2_/porous nanorods	Acetone/100 ppm	53.1	9 s/2.5 s	260 °C	<10 ppm	[131]
W_18_O_49_/Pt/porous nanospheres	Acetone/20 ppm	85	13 s/11 s (50 ppm)	180 °C	0.052 ppm	[132]
Pt-doped-3D-SnO_2_/porous hierarchical structure	Acetone/100 ppm	505.7	130 s/140 s	153 °C	<0.05 ppm	[134]
Ni doped ZnO/porous hierarchical structure	Acetone/100 ppm	68	6 s/2 s	340 °C	0.116 ppm	[135]
CuFe_2_O_4_/ α-Fe_2_O_3_/porous composite shell	Acetone/100 ppm	14	NA	275 °C	0.1 ppm	[136]
3D- WO_3_/Au/porous nano- composite	Acetone/1.5 ppm	7.6	7 s/8 s	410 °C	0.1 ppm	[137]
ZnO nanowires-loaded Sb-doped SnO_2_-ZnO/hierarchical structure	Acetone/5 ppm	12.1 & 27.8	<16 s & 32 s (res)/NA (rec)	400 °C	4.3 & 8.1 ppm	[138]
ZnO/3D-flower-like hierarchical structure	Acetone/100 ppm	18.6	7 s/NA	300 °C	NA	[139]
Au-SnO_2_/ hierarchical structure	Acetone/100 ppm	40.42	22 s/90 s	200 °C	0.445 ppm	[140]
In_2_O_3_–CuO/3D-inverse opals structure	Acetone/0.5 ppm	4.8	13 s/20 s	370 °C	0.05 ppm	[141]
SnO_2_/Sm_2_O_3_/mulberry-shaped structure	Acetone/100 ppm	41.14	NA	250 °C	0.1 ppm	[143]
WO_3_-SnO_2_/cactus like nano-composite	Acetone/600 ppm	26	NA	360 °C	NA	[144]
Cr doped WO_3_/urchin-like hollowspheres	Acetone/10 ppm	13.3	NA	250 °C	0.467 ppm	[146]
ZnO/MoS2 nanosheets/core-shell nanostructure	Acetone/5 ppm & 20 ppm (No UV and UV)	14.4 & 4.67	71 s/35 s & 56 s/69 s	300 °C & 100 °C	0.1 ppm	[147]
RGO-h-WO_3_/nano-composite	Acetone/ 200 ppm	1.5	14 s/NA	Room Temp.	<1 ppm	[148]
2D-C_3_N_4_-SnO_2_/nano-composite	Acetone/100 ppm	29	10 s/11 s	380 °C	0.067 ppm	[149]
In loaded WO_3_/SnO_2_/nano-composite	Acetone/50 ppm	66.5	11 s/5.5 s	200 °C	<1 ppm	[150]
Co_3_O_4_ nanowires–hollow carbon spheres/nano-composite	Acetone/200 ppm	23	NA	200 °C	<1 ppm	[151]
Fe_2_O_3_/In_2_O_3_/nano-composite	Acetone/100 ppm	>15	8 s/6 s	200 °C	NA	[152]
CuO-Ga_2_O_3_/nano-composite	Acetone/1.25 ppm	~1.3	NA	300 °C	0.1 ppm	[153]

NA = Not available; Temp. = Temperature; s = seconds; min = minutes.

**Table 2:** Detection concentration, response/recovery time, operating temperature (Temp.) and LODs to volatile alcohols by diverse nanostructured materials.

Materials/Nanostructure	Analyte/Concentration	Gas Response (R_air_/R_gas_)	Response/Recovery	Temp.	LOD	Ref
Sn_3_N_4_/ nanoparticles	Ethanol /100 ppm	51.3	NA	120 °C	0.07 ppm	[154]
C doped TiO_2_/nanoparticles	n-Pentanol/100 ppm	11.12	100 s/675 s	170 °C	0.5 ppm	[156]
Pr doped In_2_O_3_/nanoparticles	Ethanol/50 ppm	106	16.2 s/10 s	240 °C	<1 ppm	[157]
Au and Cl Comodified LaFeO3/nanoparticles	Ethanol/100 ppm	102.1 & 220.7	<40 s/NA	120 °C	<10 ppm	[158]
LaFe_x_O_3 −⸹_/nanocrystals	Ethanol/1000 ppm	132	1 s/1.5 s	140 °C	<50 ppm	[159]
Cl doped LaFe_x_O_3 −⸹_/nanocrystals	Ethanol/200 ppm	79.2	9 s/5 s	136 °C	<100 ppm	[160]
α-MoO_3_/nanocrystals	Ethanol/100 ppm	>55	34 s/70 s	350 °C	NA	[161]
CuO/Cu_2_O/nanocrystals	Ethanol/100 ppm	10 & 9.5	5 s/10 s & 4.1 s/10.5 s	300 °C & 275 °C	<10 ppm	[162]
Gd_1–x_Ca_x_FeO_3_/nanocrystals	Methanol/600 ppm	117.7	1 min/1.1 min	260 °C	<50 ppm	[163]
Au modified ZnO_/_nanowires	Ethanol/500 ppm	12.35	215 s/180 s	350 °C	<10 ppm	[164]
Fe_2_O_3_ nanoparticles coated SnO_2_/nanowires	Ethanol/200 ppm	57.56	300 s/100 s	300 °C	<5 ppm	[165]
In_2_O_3_ nanoparticles decorated ZnS/nanowires	Ethanol/500 ppm	>25	400 s/100 s	300 °C	<10 ppm	[166]
Sr-doped cubic In_2_O_3_/rhombohedral In_2_O_3_/nanowires	Ethanol/1 ppm	21	<1 m (both)	300 °C	0.025 ppm	[167]
Cr_2_O_3_ nanoparticles functionalized WO_3_/nanorods	Ethanol/200 ppm	5.58	51.35 s/48.65 s	300 °C	<5 ppm	[168]
ZnO/nanorods	Ethanol/100 ppm	23	26 s/43 s	Room Temp.	<5 ppm	[169]
1D-ZnO/nanorods	Ethanol/100 ppm	44.9	6 s/31 s	300 °C	<10 ppm	[170]
Pd nanoparticles decorated ZnO/nanorods	Ethanol/500 ppm	81	6 s/95 s	260 °C	<100 ppm	[171]
SnO_2_/ZnO/nanorods	Ethanol/100 ppm	18.1	2 s/38 s	275 °C	<1 ppm	[172]
rGO-WO_3_.0.33H_2_O/nanoneedles	Isopropanol/100 ppm	4.96	<90 s/NA	Room Temp.	1 ppm	[173]
Sm-doped SnO_2_/nanoarrays	Isopropanol/100 ppm	117.7	12 s/20 s	252 °C	~1 ppm	[174]
SmFeO3/nanofibers	Ethylene glycol/100 ppm	18.19	41 s/47 s	240 °C	~5 ppm	[175]
In doped NiO/nanofibers	methanol/200 ppm	10.9	273 s/26 s	300 °C	25 ppm	[176]
SiO_2_@SnO_2/_core-shell nanofibers	Ethanol/200 ppm	37	13 s/16 s	NA	NA	[177]
Yb doped In_2_O_3_/nanofibers	Ethanol/10 ppm	40 & 5	NA	Room Temp.	<1 & 5 ppm	[178]
CuO/CuCo_2_O_4_/nanotubes	n-Propanol/10 ppm	14	6.3 s/4.1 s	Room Temp.	<10 ppm	[179]
CuO-NiO/nanotubes	Glycol/100 ppm	10.35	15 s/45 s	110 °C	0.078 ppm	[180]
NiO decorated SnO_2_/vertical standing nanotubes	Ethanol/1000 ppm	123.7	10 s/58 s	250 °C	NA	[181]
Ca doped In_2_O_3_/nanotubes	Ethanol/100 ppm	183.3	2 s/56 s	240 °C	<5 ppm	[182]
Au and Ni doped In_2_O_3_/nanotubes	Ethanol/100 ppm	16.16 & 49.74	5 s/64 s & 3 s/49 s	160 °C & 220 °C	<5 ppm (for both)	[183]
W doped NiO/nanotubes	Ethanol/100 ppm	40.56	54 s/22 s	200 °C	<5 ppm	[184]
In_2_O_3_ NPs deposited TiO_2_/nanobelts	Ethanol/100 ppm	>9	6 s/3 s	100 °C	1 ppm	[185]
α-MoO_3_/nanobelts	Ethanol/800 ppm	173	<65 s/>15 s	300 °C	<50 ppm	[186]
Zn doped MoO_3/_nanobelts	Ethanol/1000 ppm	321	<121 s (for both)	240 °C	5 ppm	[187]
MOF derived Fe_2_O_3_/nanocubes	Ethanol/100 ppm	~6	<120 s/<60 s	160–230 °C	<1 ppm	[188]
In_2_O_3_/nanocubes	Ethanol/100 ppm	85	15 s/60 s	300 °C	<5 ppm	[189]
ZIF-8 derived ZnO/hollow nanocages	Ethanol/100 ppm	139.41	2.8 s/56.4 s	325 °C	0.025 ppm	[191]
ZIF-8 derived Ag functionalized ZnO/hollow nanocages	Ethanol/100 ppm	84.6	5 s/10 s	275 °C	0.0231 ppm	[192]
Cu_2_O/hollow dodecahedral nanocages	Ethanol/100 ppm	4.6	112.4 s/157.5 s	250 °C	NA	[193]
Al-doped ZnO/nanosheets	Ethanol/100 ppm	90.2	1.6 s/1.8 s	370 °C	<1 ppm	[194]
NiO NPs decorated SnO_2_/nanosheets	Ethanol/100 ppm	153	NA	260 °C	<5 ppm	[195]
CuO NPs decorated ZnO/nanosheets	Ethanol/200 ppm	97	<7 s/<40 s	320 °C	<1 ppm	[196]
SnS_2_/nanoflakes	Methanol/150 ppm	1580	67 s/5 s	Room Temp.	NA	[197]
Co doped ZnO_/_hexagonal nanoplates	Ethanol/300 ppm	570	50 s/5 s	300 °C	~50 ppm	[199]
ZIF-8 derived α-Fe_2_O_3/_ZnO/Au/nanoplates	Ethanol/100 ppm	170	4 s/5 s	280 °C	~10 ppm	[200]
ZnO/nanoplates	Ethanol/1000 ppm	8.5	154.4 s (125 ppm)/114.2 s (1500 ppm)	164 °C	NA	[201]
Zn_2_SnO_4_/nanospheres	Ethanol/50 ppm	23.4	18 s/45 s	180 °C	~5 ppm	[202]
Indium Tungsten Oxide/ellipsoidal nanospheres	Methanol/400 ppm	>5	2 s/9 s	312 °C	~20 ppm	[203]
Ag@In_2_O_3_/core-shell nanospheres	Ethanol/50 ppm	72.56	13 s/8 s	220 °C	~2 ppm	[204]
ZnSnO_3_/hollow nanospheres	n-Propanol/500 ppm	64	NA	200 °C	0.5 ppm	[205]
ZnO/hollow nanospheres	n-Butanol/500 ppm	292	36 s/9 s	385 °C	~10 ppm	[206]
α-Fe_2_O_3_/hollow nanospheres	Methanol/10 ppm	25	8 s/9 s	280 °C	1 ppm	[207]
PdO NPs modified ZnO/nanoflowers	Methanol/150 ppm	>80	18 s/52.2 s	150 °C	<0.5 ppm	[208]
NiO_/_grained nanoflowers	Ethanol/150 ppm	35	3 s/6 s	200 °C	2.6 ppm	[209]
rGO nanosheets modified NiCo_2_S_4_/nanoflowers	Ethanol/100 ppm	>2.5	4.56 s/10.38 s	100 °C	<10 ppm	[210]
Pd and rGO modified TiO_2_/nanoflowers	Ethanol/700 ppm	>64% (for both)	6.55 s/186.97 s & 75.64 s/147.16 s	90 °C	<10 ppm	[211]
Ag-functionalizedZnO/macro-/mesoporous- nanostructure	n-Butanol/100 ppm	994.8	66 s/25 s	240 °C	<1 ppm	[212]
Al-doped ZnO/macro-/mesoporous- nanostructure	n-Butanol/100 ppm	751.96	25 s/23 s	300 °C	~1 ppm	[213]
Au loaded WO_3_/mesoporous- nanostructure	n-Butanol/100 ppm	14.35	10 s/35 s	250 °C	<10 ppm	[214]
In doped ZnO/three dimensionally ordered mesoporous- nanostructure	Ethanol/100 ppm	88	25 s/10 s	250 °C	<5 ppm	[215]
Si@ZnO NPs/ mesoporous- nanostructure	Ethanol/300 ppm	62.5	0.27 min/3.5 min	400 °C	<50 ppm	[216]
Ag loaded g-C_3_N_4/_mesoporous- nanostructure	Ethanol/50 ppm	49.2	11.5 s/7 s	250 °C	<1 ppm	[217]
Pd/SnO_2/_porous- nanostructure	Ethanol/5–200 ppm	~90%	1.5 s/18 s	300 °C	<5 ppm	[218]
SnO_2_/mesoporous- nanofibers	n-Butanol/300 ppm (for both)	556.5 & 415.3	195 s/100 s & 64 s/36 s	150 °C & 200 °C	<10 ppm	[219]
TiO_2_–SnO_2_/hierarchical branched mesoporous nano- composite	Ethanol/50 ppm	40	7 s/5 s	350 °C	0.2 ppm	[220]
Co-Doped ZnO/hierarchical mesoporous- nanostructure	Ethanol/50 ppm	54	22 s/53 s	180 °C	0.0454 ppm	[221]
Fe_2_O_3_ nanorods on SnO_2_ nanospheres/hierarchical nano- composite	Ethanol/100 ppm	23.512	5 s/12 s	320 °C	<50 ppm	[222]
MoO_3_-mixed SnO_2_/hierarchical nanostructure	Ethanol/100 ppm	714	1 s (for all)/357 s, 8 s and 85 s	260 °C	<10 ppm	[223]
In_2_O_3_ Nanoparticles Decorated ZnO/hierarchical nanostructure	n-Butanol/100 ppm	218.3	5 s/12 s	260 °C	Down to sub-ppm	[224]
SnO_2_–Si-NPA/honeycomb like nanostructure	Ethanol/50 ppm	7.7	10 s/9 s	320 °C	<10 ppm	[225]
SnO_2_/rambutan-like hierarchical nanostructure	n-Butanol/100 ppm	44.3	8 s/5 s	140 °C	<20 ppm	[226]
SnO_2_/raspberry-like hollow nanostructure	n-Butanol/100 ppm	303.49	163 s/808 s	160 °C	1 ppm	[228]
SnO_2_/snowflake-like hierarchical nanostructure	Ethanol/250 ppm	~55	6 s/7 s	400 °C	NA	[229]
SnO_2_/pentagonal-cone assembled with nanorods	Ethanol/200 ppm	98	11 s/18 s	220 °C	1 ppm	[231]
ZIF-8 derived ZnO/neck- connected nanostructure films	Ethanol/50 ppm	124	120 s/70 s	375 °C	0.5 ppm	[232]
LaMnO_3_@ZnO/nano- composite	n-Butanol /100 ppm	6	8 s/17 s	300 °C	NA	[233]
SnO_2_-Pd-Pt- In_2_O_3/_nano- composite	Methanol/100 ppm	320.73	32 s/47 s	160 °C	0.1 ppm	[234]
RGO-SnO_2_/nano- composite	Ethanol /100 ppm	43	8 s/NA	300 °C	~5 ppm	[235]
ZnO:Fe/nano-composite films	Ethanol/100 ppm	61 & 36	1.1 s/1.45 s & 0.23 s/0.34 s	250 °C & 350 °C	~10 ppm	[237]
g-C_3_N_4_-SnO_2_/nano- composite	Ethanol/500 ppm	240	15 s/38 s	300 °C	~50 ppm	[238]
Co_3_O_4_ nanosheet array-3D carbon foam/ nano- composite	Ethanol/100 ppm	10.4	45 s/140 s	100 °C	0.2 ppm	[239]

NA = Not available; Temp. = Temperature; s = seconds; min = minutes.

**Table 3:** Detection concentration, response/recovery time, operating temperature (Temp.) and LODs to volatile organic aldehyde gas by diverse nanostructured materials.

Materials/Nanostructure	Analyte/Concentration	Gas Response (R_air_/R_gas_)	Response/Recovery	Temp.	LOD	Ref
p-CuO/n-SnO_2_/core-shell nanowires	Formaldehyde/50 ppm	2.42	52 s/80 s	250 °C	<1.5 ppm	[247]
ZnO/meso-structured nanowires (under UV)	Formaldehyde/50 ppm	1223%	NA	Room Temp.	0.005 ppm	[248]
RGO coated Si/nanowires	Formaldehyde/10 ppm	6.4	30 s/10 s	300 °C	0.035 ppm	[249]
Co doped In_2_O_3_/nanorods	Formaldehyde/10 ppm	23.2	60 s/120 s	130 °C	1 ppm	[250]
Ag-functionalized Ni-doped In_2_O_3_/nanorods	Formaldehyde/100 ppm	123.97	1.45 s/58.2 s	160 °C	<2.5 ppm	[251]
Ag doped LaFeO_3_/nanofibers	Formaldehyde/5 ppm	4.8	2 s/4 s	230 °C	~5 ppm	[253]
Co_3_O_4_-ZnO/core-shell nanofibers	Formaldehyde/100 ppm	>5	6 s/9 s	220 °C	<10 ppm	[254]
WO_3_/ZnWO_4/_nanofibers	Formaldehyde/5 ppm	44.5	12 s/14 s	220 °C	1 ppm	[255]
Pr-doped BiFeO_3/_hollow nanofibers	Formaldehyde/50 ppm	17.6	17 s/19 s	190 °C	5 ppm	[256]
Ca doped In_2_O_3_/nanotubes	Formaldehyde/100 ppm	116	1 s/328 s	130 °C	0.06 ppm	[257]
Er-doped SnO_2_/nanobelt	Formaldehyde/100 ppm	9	17 s/25 s	230 °C	0.141 ppm	[258]
Pt-decorated MoO_3_/nanobelt	Formaldehyde/100 ppm	~25%	17.8 s/10.5 s	Room Temp.	1 ppm	[259]
ZnSnO_3_/multi-shelled nanocubes	Formaldehyde/100 ppm	37.2	1 s/59 s	220 °C	<10 ppm	[260]
ZnSn(OH)_6_/multi-shelled nanocubes	Formaldehyde/100 ppm	56.6	1 s/89 s	60 °C	1 ppm	[261]
MOF-derived Co_3_O_4_/CoFeO_4_/double-shelled nanocubes	Formaldehyde/10 ppm	12.7	4 s/9 s	139 °C	0.3 ppm	[262]
WO_x_ clusters decorated In_2_O_3_/nanosheets	Formaldehyde/100 ppm	~25	1 s/67 s	170 °C	0.1 ppm	[263]
SnO_2_/nanosheets	Formaldehyde/200 ppm	207.7	30 s/57 s	200 °C	0.1 ppm	[264]
Au atom dispersed In_2_O_3/_nanosheets	Formaldehyde/50 ppm	85.67	25 s/198 s	100 °C	0.00142 ppm	[266]
SnS_2/_nanoflakes film	Formaldehyde/NA	NA	NA	210 °C	0.001/0.02 ppm	[268]
0D ZnS/nanospheres and nanoparticles	Formaldehyde/100 ppm	95.4 & 68.2	11 s/8 s	295 °C	~5 & 10 ppm	[269]
Ag doped Zn_2_SnO_4_/SnO_2/_hollow nanospheres	Formaldehyde/50 ppm	60	9 s/5 s	140 °C	5 ppm	[270]
SnO_2_/nanoflowers (hierarchical)	Formaldehyde/100 ppm	34.6	64 s/10 s	300 °C	5 ppm	[272]
Sn_3_O_4_/rGO_/_nanoflower (hetero- structure)	Formaldehyde/100 ppm	44	4 s/125 s	150 °C	1 ppm	[273]
Au-loaded In_2_O_3_/porous hierarchical nanocubes	Formaldehyde/100 ppm	37	3 s/8 s	240 °C	10 ppm	[274]
Ag-loaded ZnO/porous hierarchical nano- composite	Formaldehyde/100 ppm	170.42	12 s/90 s	240 °C	1 ppm	[275]
Pd–WO_3_/m-CN/mesoporous nanocubes	Formaldehyde/25 ppm	24.2	6.8 s/4.5 s	120 °C	1 ppm	[276]
GO/SnO_2_/2D mesoporous nanosheets	Formaldehyde/100 ppm	2275.7	81.3 s/33.7 s	60 °C	0.25 ppm	[277]
ZnSnO_3/_2D mesoporous nanostructure	Formaldehyde/100 ppm	45.8	3 s/6 s	210 °C	0.2 ppm	[278]
LaFeO_3_/porous hierarchical nanostructure	Formaldehyde/50 ppm	116	7 s/24 s	125 °C	0.05 ppm	[279]
Bi doped Zn_2_SnO_4_/SnO_2_/porous nanospheres	Formaldehyde/50 ppm	23.2	16 s/9 s	180 °C	10 ppm	[280]
ZnO/porous nanoplates	Formaldehyde/100 ppm	12	80 s/60 s	240 °C	10 ppm	[281]
Au@ZnO/mesoporous nanoflowers	Formaldehyde/100 ppm	45.28	NA	220 °C	NA	[282]
Zn_2_SnO_4_/SnO_2_/hierarchical octahedral nanostructure	Formaldehyde/100 ppm	>60	76 s/139 s (for 20 ppm)	200 °C	2 ppm	[284]
SnO_2_ nanofiber/nanosheet/hierarchical nanostructure	Formaldehyde/100 ppm	57	4.7 s/11.6 s	120 °C	~0.5 ppm	[286]
In_2_O_3_@SnO_2_/hierarchical nano- composite	Formaldehyde/100 ppm	180.1	3 s/3.6 s	120 °C	0.01 ppm	[287]
SnO_2_/cedar like hierarchical nano-micro structure	Formaldehyde/100 ppm	13.3	<1 s/13 s	200 °C	~5 ppm	[288]
In_2_O_3/_urchin like hollow nanostructure	Formaldehyde/1 ppm	20.9	46 s/90 s	140 °C	0.05 ppm	[289]
SnO_2_/Butterfly like hierarchical nanostructure	Acetaldehyde/100 ppm	178.3	28 s/58 s	243 °C	<0.5 ppm	[290]
SnO_2_/hollow hexagonal prisms	Formaldehyde/2 ppm	882	19 s/NA	120 °C	<2 ppm	[291]
NiO/NiFe_2_O_4_/nano- tetrahedrons composite	Formaldehyde/100 ppm	22.5	9 s/3 s	240 °C	0.2 ppm	[292]
VG/SnO_2_/nano- composite	Formaldehyde/5 ppm	>5	46 s/95 s	Room Temp.	0.02 ppm	[293]

NA = Not available; Temp. = Temperature; s = seconds; min = minutes.

**Table 4:** Detection concentration, response/recovery time, operating temperature (Temp.) and LODs to volatile organic amine gas by diverse nanostructured materials.

Materials/Nanostructure	Analyte/Concentration	Gas Response (R_air_/R_gas_)	Response/Recovery	Temp.	LOD	Ref
Co_3_O_4_/ZnO/hybrid nanoparticles	Triethylamine/200 ppm	282.3	25 s/36 s	280 °C	~10 ppm	[296]
Ho-doped SnO_2_/nanoparticles	Triethylamine/50 ppm	12	2 s/2 min	175 °C	~5 ppm	[297]
CuCrO_2_/nanoparticles	Triethylamine/100 ppm	~5	90 s/120 s	140 °C	~10 ppm	[298]
Ag/Pt/W_18_O_49_/nanowires	Triethylamine/50 ppm	813	15 s/35 s (for 2 ppm)	240 °C	0.071 ppm	[299]
1D SnO_2_ coated ZnO/hybrid nanowires	n-Butylamine/10 ppm	7.4	40 s/80 s	240 °C	~1 ppm	[300]
V_2_O_5_ -decorated α-Fe_2_O_3_/nanorods	Diethylamine/100 ppm	8.9	2 s/40 s	350 °C	~5 ppm	[301]
Au NPs decorated WO_3_/nanorods	Trimethyl-amine/100 ppm	76.7	6 s/7 s	280 °C	~5 ppm	[302]
Ag NPs decorated α-MoO_3_/nanorods	Triethylamine/100 ppm	408.6	3 s/107 s	200 °C	0.035 ppm	[303]
Cr dopedα-MoO_3_/nanorods	Triethylamine/100 ppm	150.25	7 s/80 s	200 °C	~1 ppm	[304]
Acidic α-MoO_3_/nanorods	Triethylamine/100 ppm	101.74	4 s/88 s	300 °C	0.1 ppm	[305]
Au@SnO_2_/α-Fe_2_O_3_/core-shell nanoneedles on alumina tubes	Triethylamine/100 ppm	39	4 s/203 s	300 °C	~2 ppm	[307]
Al_2_O_3_/α-Fe_2_O_3_/nanofibers	Triethylamine/100 ppm	15.19	1 s/17 s	250 °C	~0.5 ppm	[308]
In_2_O_3_/hierarchical nanofibers (with nanoparticles)	Triethylamine/50 ppm	87.8	148 s/40 min	40 °C	~5 ppm	[309]
Nb doped TiO_2_/nanotubes	Dimethyl-amine/50 ppm	9.1	≥300 s (for both)	300 °C	~5 ppm	[312]
Au NPs decorated MoO_3_/nanobelts	Trimethyl-amine/50 ppm	70	6 s/9 s	280 °C	~5 ppm	[313]
W doped MoO_3_/nanobelts	Trimethyl-amine/50 ppm	13.8	6 s/11 s	280 °C	~5 ppm	[314]
RuO_2_ NPs decorated MoO_3_/nanobelts	Triethylamine/10 ppm	75	2 s/10 s	260 °C	~1 ppm	[315]
ZnO-SnO_2_/nanobelts	Triethylamine/100 ppm	9.9	1.8 s/18 s	220 °C	~1 ppm	[316]
In_2_O_3_/nanocubes	Triethylamine/100 ppm	175	11 s/14 s	180 °C	~10 ppm	[317]
WO_3_/nanosheets	Triethylamine/1000 ppm	~14	NA	Room Temp.	~5 ppm	[318]
Au@ZnO- SnO_2_/nanosheets	Triethylamine/100 ppm	115	7 s/30 s	300 °C	~2 ppm	[319]
TiO_2_ NPs decorated CuO/nanosheets	Triethylamine/5 ppm	12.7	45 s/202 s	160 °C	0.5 ppm	[320]
Rh-SnO_2_/nanosheets	Triethylamine/100 ppm	607.2	49 s/24 s	325 °C	~1 ppm	[321]
Ag modified Zn_2_SnO_4_/hexagonal nanoflakes- hollow octahedron	Triethylamine/50 ppm	83.6	<1 s/20 s	220 °C	~1 ppm	[322]
Zn_2_SnO_4_- doped SnO_2_/hollow nanospheres	Phenylamine/50 ppm	4.53	10 s/12 s	300 °C	~1 ppm	[323]
CeO_2_-SnO_2_/nanoflowers	Triethylamine/200 ppm	252.2	NA	310 °C	~20 ppm	[324]
WO_3_-SnO_2_/mesoporous nanostructure	Triethylamine/50 ppm	87	6 s/7 s	220 °C	~1 ppm	[325]
CuO/porous particles with diverse morphologies	Triethylamine/100 ppm	5.6–102	<40 s/<260 s	230 °C	~5 ppm	[326]
In_2_O_3_/mesoporous nanocubes	Trimethyl-amine/10 ppm	57	4 s/11 s	160 °C	~5 ppm	[327]
CeO_2_/porous nanospheres	Triethylamine/100 ppm	4.67	13 s/<230 s	Room Temp.	~5 ppm	[328]
Au decahedrons-decorated α-Fe_2_O_3_/porous nanorods	Triethylamine/50 ppm	17	12 s/18 s	40 °C	1 ppm	[329]
ZnCo_2_O_4_/porous nano- structures	Triethylamine/100 ppm	14	7 s/57 s	200 °C	~5 ppm	[330]
NiCo_2_O_4_/porous nanoplates	Triethylamine/10 ppm	2.58	<33 s/42 s	220 °C	0.5 ppm	[331]
SnO_2_/porous thin films	Triethylamine/10 ppm	150.5	53 s/120 s	Room Temp.	0.11 ppm	[332]
Fe_2_O_3_/ZnFe_2_O_4_/porous nano- composite	Triethylamine/20 ppm	60.24	2 s/7 s	300 °C	0.2 ppm	[333]
Au-Modified ZnO/porous hierarchical nanosheets	Trimethyl-amine/30 ppm	65.8	3.3 s/64 s	260 °C	0.01 ppm	[334]
α-Fe_2_O_3_/snowflake-like hierarchical nanostructure	Trimethyl-amine/100 ppm	10.9	0.9 s/1.5 s	260 °C	~5 ppm	[335]
Zn_2_SnO_4_–ZnO/hierarchical nano- composite	Triethylamine/100 ppm	175.5	12 s/25 s	200 °C	0.4 ppm	[336]
MoS_2_/GO/3D hierarchical nano- composite	Triethylamine/100 ppm	192	20 s/18 s	260 °C	1 ppm	[337]
Au NPs decorated Co_3_O_4_/hierarchical nanochains	Triethylamine/300 ppm	>40	94 s/100 s	210 °C	~10 ppm	[338]
WO_3_/hierarchical flower like spheres	Triethylamine/10 ppm	11.6	3 s/55 s	205 °C	0.083 ppm	[339]
ZnO/Au/hemishperical nanostructure	Triethylamine/100 ppm	104.8	5 s/2 s	260 °C	~10 ppm	[340]
SnO_2_/Au/Fe_2_O_3_/nanoboxes	Triethylamine/100 ppm	126.84	7 s/10 s	240 °C	0.05 ppm	[341]
Au decorated ZnO/nest-like nanostructure	Triethylamine/200 ppm	625	4 s/26 s	320 °C	1 ppm	[342]
Pd doped ZnO/agaric like nanostructure	Aniline/100 ppm	182	29 s/23 s	280 °C	0.5 ppm	[343]
Co_3_O_4_@MnO_2_/shish-kebab like nanostructure	Triethylamine/100 ppm	9.13	93 s/92 s	250 °C	~10 ppm	[344]
Au@ZnO/core-shell nanostructure	Triethylamine/5 ppm	12.2%	27 s/46 s	50 °C	~1 ppm	[345]
Au/Co_3_O_4_/W_18_O_49_/hollow composite nanospheres	Triethylamine/2 ppm	16.7	9 s/14 s	270 °C	0.081 ppm	[347]
α-Fe_2_O_3_@α- MoO_3_/nano- composite	Triethylamine/50 ppm	76	4 s/370 s	280 °C	~2 ppm	[348]
rGO decorated W doped BiVO_4_/hierarchical nano- composite	Trimethyl-amine/20 ppm	12.8	16 s/NA	135 °C	0.63 ppm	[350]
Au@MoS_2_/nano- composite	Triethylamine/50 ppm	44	9 s/91 s	30 °C	~2 ppm	[351]

NA = Not available; Temp. = Temperature; s = seconds; min = minutes.

**Table 5:** Detection concentration, response/recovery time, operating temperature (Temp.), and LODs to volatile hydrocarbons gases by diverse nanostructured materials.

Materials/Nanostructure	Analyte/Concentration	Gas Response (R_air_/R_gas_)	Response/Recovery	Temp.	LOD	Ref
Ag-LaFeO_3_/nanoparticles	Xylene/5 ppm	36.2	114 s/55 s	99 °C	<1 ppm	[352]
Ag-LaFeO_3_/nanoparticles	Xylene/10 ppm	16.76	68 s/36 s	125 °C	0.2 ppm	[353]
Au-ZnO/nanoparticles	Xylene/100 ppm	92	4 s/6 min	377 °C	NA	[354]
cobalt porphyrin (CoPP)-functionalized TiO_2_/nanoparticles	Benzene, Toluene and Xylene (BTX)/10 ppm	>5	40 s/80 s	240 °C	0.005 ppm	[355]
In-doped ZnO/Quantum dots	Acetylene/10 ppm	19.3	~100 s/NA	400 °C	0.1 ppm	[356]
Metal organic Frameworks/nanocrystals	Benzene, Toluene, Ethyl benzene and Xylene (BTEX)/50 ppm	>20	NA	Room Temp.	0.4 ppm	[357]
α-Fe_2_O_3_/SnO_2_/nanowire arrays	Toluene/100 ppm	49.7	20 s/15 s	90 °C	~50 ppm	[358]
Pt NPs sensitizedSi NW-TeO_2_/nanowires	Toluene/50 ppm	45	20 s/500 s	200 °C	~10 ppm	[359]
CoPP-ZnO/nanorods	Toluene/10 ppm	>2.5	NA	NA	0.002 ppm	[361]
α-MoO_3_/nanoarrays	Xylene/100 ppm	19.2	1 s/≤20 s	370 °C	~10 ppm	[362]
Y doped α-MoO_3_/nanoarrays	Xylene/100 ppm	28.3	1 s/~15 s	370 °C	~5 ppm	[363]
MOF-driven metal- embedded metal oxide (Pd@ZnO- WO_3_)/nanofibers	Toluene/1 ppm	22.22	<20 s/NA	350 °C	0.1 ppm	[364]
V_2_O_5_/nanofibers	Xylene/500 ppm	191	80 s/50 s (100 ppm)	Room Temp.	~5 ppm	[365]
Pd functionalized SnO_2_/nanofibers	Butane/3000 ppm	47.58	3.20 s/6.28 s	260 °C	~10 ppm	[366]
Pt-decorated CNTs/nanotubes	Toluene/5 ppm	5.06	90 s/520 s	150 °C	~1 ppm	[367]
3D TiO_2_/G-CNT/nanotubes	Toluene/500 ppm	42.9%	9–11 s (for both)	Room Temp.	0.4 ppm	[368]
NiCo_2_O_4_/nanotubes (hierarchical)	Xylene/100 ppm	9.25	20 s/9 s	220 °C	~1 ppm	[369]
Fe doped MoO_3_/nanobelts	Xylene/100 ppm	6.1	20 s/75 s	206 °C	~5 ppm	[370]
Au decorated ZnO/In_2_O_3/_belt-tooth nanostructure	Acetylene/100 ppm	5	8.5 s/NA	90 °C	~25 ppm	[371]
ZnO/ZnCo_2_O_4_/hollow nanocages	Xylene/100 ppm	34.26	NA	320 °C	0.126 ppm	[372]
Au functionalizedWO_3_·H_2_O/nanosheets	Toluene/100 ppm	50	2 s/9 s	300 °C	~10 ppm	[373]
Nb-doped NiO/nanosheets	Xylene/100 ppm	335.1	63 s/66 s	370 °C	0.002 ppm	[375]
CdO/hexagonal nanoflakes	liquefied petroleum gas (LPG)/500 ppm	~27.5	8.6 s/10 s	270 °C	~10 ppm	[376]
ZnO-CeO_2_/triangular nanoflakes	BTEX/50 ppm	10–21	8 s/10 s	200 °C	0.01 ppm	[377]
ZnFe_2_O_4_/nanospheres	Toluene/100 ppm	9.98	18.14 s/29.2 s	300 °C	~1 ppm	[378]
Pt doped CoCr_2_O_4_/hollow nanospheres	Xylene/5 ppm	559	300 s/600 s	275 °C	0.0187 ppm	[379]
Pd-SnO_2_/nanoporous composite	Methane/3000 ppm	17.6	3 s/5 s	340 °C	~100 ppm	[380]
Co_3_O_4_–TiO_2/_mesoporous hierarchical nanostructure	Xylene/50 ppm	113	130 s/150 s	115 °C	~5 ppm	[381]
Au loaded MoO_3_/hollow nanospheres (hierarchical)	Toluene and Xylene/100 ppm	17.5 & 22.1	19 s/6 s & 1.6 s/2 s	250 °C	0.1 & 0.5 ppm	[382]
Pt-SnO_2/_hollow nanospheres (hierarchical)	Methane/250 ppm	4.88 & 4.33	NA	300 °C & 340 °C	~25 ppm	[383]
NiO/NiMoO_4_/hierarchical nanospheres	*p*-Xylene/5 ppm	101.5	10-50 s/20-200 s	375 °C	0.02 ppm	[384]
Co_3_O_4_/hierarchical nanostructure	Toluene/200 ppm	8.5	10 s/30 s	180 °C	~5 ppm	[385]
WO_3_/hierarchical nanostructure	Acetylene/200 ppm	32.31	12 s/17 s	275 °C	<5 ppm	[386]
PbS NPs decorated CdO/necklace like nanobeads	LPG/1176 ppm	~50	148 s/142 s	Room Temp.	~300 ppm	[387]
Au loaded TiO_2_/hedgehog-like nanostructure	Xylene/100 ppm	6.49	5 s/2 s	375 °C	~2 ppm	[388]
Pd/PdO/S-SnO_2_/nano- composite film	methane/300 ppm	12.3	8 s/12 s	240 °C	<50 ppm	[390]
rGO/Co_3_O_4/_nano- composite	Toluene/5 ppm	11.3	NA	110 °C	≥0.5ppm	[391]
WO_3_ decorated TiO_2_ NPs/nano- composite	Xylene/10 ppm	92.53	410 s/2563 s	160 °C	1 ppm	[392]
BGQD/Ag–LaFeO_3_/nano- composite	Benzene/1 ppm	17.5	NA	65 °C	<1 ppm	[393]
Ag/Bi_2_O_3_/nano- composite	Toluene/50 ppm	89.2%	NA	Room Temp.	~10 ppm	[394]
AgO loaded LaFeO_3_/nano- composite	Acetylene/100 ppm	60	6.1 min/4.7 min	200 °C	~5 ppm	[395]
CuO NPs-Ti_3_C_2_TxMXene/nano- composite	Toluene/50 ppm	11.4	270 s/10 s	250 °C	0.32 ppm	[396]
Graphene/SnO_2_ NPs nano-composite	BTX/0.2–11 ppm	1–28	NA	RT and 250 °C	~0.2 ppm	[397]

NA = Not available; Temp. = Temperature; s = Seconds; min = minutes.

## Data Availability

No new data were created or analyzed in this study. Data sharing is not applicable to this article.
